# Missing Rings in *Pinus halepensis* – The Missing Link to Relate the Tree-Ring Record to Extreme Climatic Events

**DOI:** 10.3389/fpls.2016.00727

**Published:** 2016-05-31

**Authors:** Klemen Novak, Martin de Luis, Miguel A. Saz, Luis A. Longares, Roberto Serrano-Notivoli, Josep Raventós, Katarina Čufar, Jožica Gričar, Alfredo Di Filippo, Gianluca Piovesan, Cyrille B. K. Rathgeber, Andreas Papadopoulos, Kevin T. Smith

**Affiliations:** ^1^Department of Geography and Regional Planning – Instituto de Investigación en Ciencias Ambientales, University of ZaragozaZaragoza, Spain; ^2^Department of Ecology, University of AlicanteAlicante, Spain; ^3^Department of Wood Science and Technology, Biotechnical Faculty, University of LjubljanaLjubljana, Slovenia; ^4^Slovenian Forestry InstituteLjubljana, Slovenia; ^5^Dendrology Lab, Department of Agriculture and Forestry Science (DAFNE), University of TusciaViterbo, Italy; ^6^LERFoB, INRA, AgroParisTechNancy, France; ^7^Department of Forestry and Natural Environmental Management, T.E.I. Stereas ElladasKarpenissi, Greece; ^8^USDA Forest Service, Northern Research Station, DurhamNH, USA

**Keywords:** Aleppo pine, tree rings, climate–growth relationship, climate, extreme growth event, Mediterranean

## Abstract

Climate predictions for the Mediterranean Basin include increased temperatures, decreased precipitation, and increased frequency of extreme climatic events (ECE). These conditions are associated with decreased tree growth and increased vulnerability to pests and diseases. The anatomy of tree rings responds to these environmental conditions. Quantitatively, the width of a tree ring is largely determined by the rate and duration of cell division by the vascular cambium. In the Mediterranean climate, this division may occur throughout almost the entire year. Alternatively, cell division may cease during relatively cool and dry winters, only to resume in the same calendar year with milder temperatures and increased availability of water. Under particularly adverse conditions, no xylem may be produced in parts of the stem, resulting in a missing ring (MR). A dendrochronological network of *Pinus halepensis* was used to determine the relationship of MR to ECE. The network consisted of 113 sites, 1,509 trees, 2,593 cores, and 225,428 tree rings throughout the distribution range of the species. A total of 4,150 MR were identified. Binomial logistic regression analysis determined that MR frequency increased with increased cambial age. Spatial analysis indicated that the geographic areas of south-eastern Spain and northern Algeria contained the greatest frequency of MR. Dendroclimatic regression analysis indicated a non-linear relationship of MR to total monthly precipitation and mean temperature. MR are strongly associated with the combination of monthly mean temperature from previous October till current February and total precipitation from previous September till current May. They are likely to occur with total precipitation lower than 50 mm and temperatures higher than 5°C. This conclusion is global and can be applied to every site across the distribution area. Rather than simply being a complication for dendrochronology, MR formation is a fundamental response of trees to adverse environmental conditions. The demonstrated relationship of MR formation to ECE across this dendrochronological network in the Mediterranean basin shows the potential of MR analysis to reconstruct the history of past climatic extremes and to predict future forest dynamics in a changing climate.

## Introduction

The reports of the Intergovernmental Panel on Climate Change (IPCC, 2013) and the European Environmental Agency (Füssel, 2012) indicate substantial warming and increased frequency and intensity of drought, heat waves, and uncertainty of regional and seasonal climatic variability across most of the Mediterranean area (FAO and Plan Bleu, 2013). The impacts of climate change are strongly related to the increase in frequency and severity of extreme climatic events (ECE; e.g., IPCC, 2013; Panayotov et al., 2013). Under such conditions, trees may decline in annual growth (Haavik et al., 2015), become more vulnerable to secondary damage from attacks by insect pests (Esper et al., 2007a; Sangüesa-Barreda et al., 2014; Robson et al., 2015) and fungal diseases (Cherubini et al., 2002), and experience higher rates of mortality (Camarero et al., 2015).

Extreme events are difficult to define, because they can vary depending on discipline (e.g., hydrology, climatology, agriculture, forestry), type of events (e.g., heat waves, drought, precipitation, strong winds), and/or climate area (e.g., Mediterranean, Continental, Alpine). Sarewitz and Pielke (2001) defined an extreme event as “an occurrence that, with respect to some class of occurrences, is notable, rare, unique, profound or otherwise significant in terms of its impacts, effects or outcome.” Smith (2011) suggested the need to define extreme events synthetically, from both the “driver” (occurrence) and “response” (effect) perspectives.

Similarly, the impacts of ECE on forests are also difficult to evaluate, as they are low in frequency and generally local in occurrence. Dendrochronology is a useful tool to analyze their impacts because it can operate on wide spatial and temporal scale. It is based on analyses of annual tree-ring widths, and its characteristics which vary due to combined influence of various ecological factors and climatic conditions affecting tree growth, and therefore they can be considered as natural archives of past events with high (i.e., annual) resolution (Fritts, 2001). Tree-ring widths and density variations have been widely used to reconstruct past temperature and precipitation (Hughes, 2002).

The anatomical structure of tree rings often contain indications of abrupt change in temperature, precipitation regime, or due to natural disturbances (Panayotov et al., 2013). Such structures in tree rings include intra-annual density fluctuations (Novak et al., 2013a; Campelo et al., 2015), resin canals (Rigling et al., 2003; Novak et al., 2013b), and proportions of earlywood and latewood (Lebourgeois, 2000; Novak et al., 2013b). Anatomical features provide a promising approach to better understand the influence of climate on tree rings (Fonti et al., 2010) and can complement traditional analysis of the climatic signal obtained from tree-ring width (Lebourgeois, 2000; Novak et al., 2013b).

Missing rings (MR) are detected through comparison of crossdated series of tree rings contained in analyzed samples. Rarely is a tree ring missing from all woody parts of the plant (Novak et al., 2011; Wilmking et al., 2012; Liang et al., 2014).

The occurrence of MR is related to the annual pattern of cambial activity (cell division) and cell differentiation which varies across the species’ distribution. In Mediterranean areas the cambium generally stops dividing in summer as a consequence of drought (e.g., de Luis et al., 2011a) and in winter at the temperature limited sites (e.g., Liphschitz et al., 1984; Liphschitz and Lev-Yadun, 1986). Under favorable growing conditions, the cambium may be active almost throughout the entire year (de Luis et al., 2007, 2011a,b). In warm Mediterranean sites, prolonged dry winter periods can stop cambial activity, which resumes with increasing water availability (Camarero et al., 2010). Under particularly adverse conditions through the entire growing season, no xylem may be produced in parts of the stem, resulting in a MR (Novak et al., 2011, 2016).

Missing rings are usually considered as a “problem” that hampers the correct dating of tree rings (Grundmann et al., 2008; Rutherford and Mann, 2014). Conceptually, analysis of MR poses a challenge in that they are absent from direct observation. To our knowledge, MR have not been used as a “proxy” or temporal marker to analyze environmental processes.

Missing rings indicate an absence of wood production by the vascular cambium for one or more particular years (and parts of a tree), due to different stresses, such as unfavorable climatic events, competition (Lorimer et al., 1999; Parent et al., 2002), pests (Sangüesa-Barreda et al., 2014), or diseases (Cherubini et al., 2002). They are common in different species and in different environments (Jonsson et al., 2002; Wilmking et al., 2012; Dulamsuren et al., 2013; Liang et al., 2014). MR frequently occur in conifers and particularly in trees growing in the Mediterranean basin, with an increased frequency of occurrence in recent years noted for *Pinus halepensis* (Raventós et al., 2001; Novak et al., 2011, 2013b).

Missing rings are like extreme events, relatively rare in space and time. A typical dendroclimatic analysis may involve the collection of two increment core samples from each of 15 trees per site. The results of observations from 30 samples is likely not sufficient for the robust estimation of the frequency of occurrence of MR at the sampling site. The combined effect on MR occurrence due to the age and size of trees, known as the biological trend, complicates determination of the influence of climate on tree-ring growth. The binary character of the occurrence of a MR (presence, absence) is a challenge for standardization of the biological trend in MR frequency as well as to use MR series to identify climatic signals using standard dendrochronological techniques. Different statistical approaches have been proposed so far (Campelo et al., 2007, 2015; Novak et al., 2013b; Zalloni et al., 2016), but the most appropriate tests for this type of data are not yet confirmed.

*Pinus halepensis* is the most widespread Mediterranean pine tree species. Its spatial distribution has the potential to produce a useful network of sites with different climatic conditions. MR in *Pinus halepensis* can be evaluated across wide spatial and temporal gradients and related to ECE. Therefore, to determine the potential of MR as markers of ECE in Mediterranean forests, the objectives of this study are:

(1) To analyze the frequency of MR using a dendrochronological network across the distribution area of *Pinus halepensis*;
(2) To implement new statistical techniques to identify age-related trends in binomial dendrochronological data and climatic signals associated with the occurrence of MR as an example of low frequency events;
(3) To analyze climatic thresholds (the extremes of temperatures and precipitation) which promote and trigger the “formation” of MR, and their operation across the temporal scale; and
(4) To estimate the frequencies of MR across the species distribution as related to the extremes of temperatures and precipitation.

## Materials and Methods

### Dendrochronological Network for *Pinus halepensis*

**FIGURE 1:**
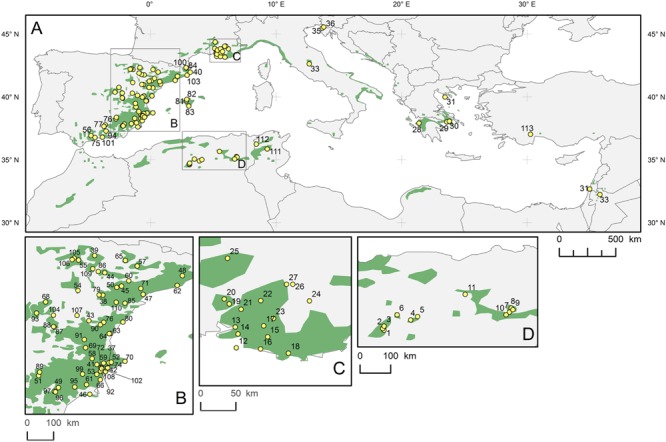
**Distribution range of *Pinus halepensis* (green surface) in the Mediterranean basin **(A)**; details in **(B–D)**, and locations of the study sites (yellow dots with numbers).** The base map was obtained from the European Forest Genetic Resources Programme (http://www.euforgen.org/distribution-maps/).

*Pinus halepensis* grows throughout the entire Mediterranean area as shown on the distribution map (**Figure 1**). The network of sites selected for dendrochronological sampling consisted of newly collected and archived tree-ring series from 113 sites (Supplementary Table S1), covering the area extending from 32.23° to 45.67°N latitude, 1.41°W to 36.17°E longitude, and altitudes from 15 to 1676 m a. s. l.

Monthly total precipitation and monthly mean temperature for 1,068 spatial grid points within the distribution range of *Pinus halepensis* for the 1901–2014 period were obtained from the Climatic Research Unit of the University of East Anglia. We used the CRU 3.22 dataset of 0.5° grid resolutions (Harris et al., 2014), and assigned the climatic data from the nearest grid point to each sampling site.

The tree-ring dataset was derived from a total of 2,593 increment cores collected from 1,509 trees of the dendrochronological network. Tree selection, core sampling, and processing were performed using standard dendrochronological techniques (Cook and Kairiukstis, 1990; Speer, 2010). Tree-ring widths were measured under a stereo microscope with an accuracy of 0.01 mm, using the TSAP-Win program and LINTAB^TM^ 5 measuring device (Rinntech^®^, Heidelberg, Germany^1^). Globally, a total of 225,428 tree rings were dated and measured. Tree-ring series were visually and statistically crossdated and compared with each other by calculating the *t*-value after Baillie and Pilcher (1973) using TSAP-Win. The quality of crossdating was verified using the dendrochronology program library in R (dplR; Bunn, 2008). A MR was determined for each crossdated ring-width series by scoring every year as either being missing (value of 1) or present (value of 0). This process accommodated analysis of a ring missing from a series.

### Age Effect and Standardizing Procedures

The term “cambial age” refers to the number of years that the vascular cambium has produced annual rings prior to and including the ring being examined. For example, the first tree ring produced outside of the pith has a cambial age of 1; the tenth ring has an age of 10, and so on. Consequently, rings produced in a single calendar year by trees of different ages will differ in cambial age. The effect of cambial age on the occurrence of MR was estimated by binomial logistic regression modeling of the complete collection of MR series. To construct this model, the dependent variable was the series of all individual MR values (0 or 1) and the independent variable was their cambial age. The resulting regression line provided the estimated cambial age-related trend in MR frequency. Then, each MR series was standardized by calculating the ratio between the observed MR value and the value predicted by the trend model at that cambial age. The mean MR series for each site was calculated from the individual standardized MR values.

### Climate Conditions Promoting MR Occurrence

Climatic conditions associated to each individual tree ring formed through the 1901–2014 period were obtained from CRU 3.22 dataset. For each individual tree ring, 16 pairs of monthly climate variables were calculated (sum of precipitation and mean temperatures from the previous September to current December). In addition, for each monthly climate variable, the mean of each preceding 2-months, 3-months and so on up to 60 months window were calculated to test the persistence of climatic effects on MR frequency over longer periods of time. Then, a total of 960 different values of precipitation and 960 of temperature (16 months*60 months period) were calculated and associated to each individual tree ring.

Logistic regression models (LRM) were used to determine the relationship between MR frequency (dependent variable) and total precipitation, mean temperature, and their interaction (independent variables). A set of LRM was constructed to identify the combination of climatic factors which explain better the observed frequency of MR across the network. Globally a total of 921,600 LRM were constructed to consider all paired combinations of different variables of precipitation and temperature. Goodness-of-fit were compared by the coefficient of determination (*r*^2^) of the various models.

For each calculation of LRM hierarchical cluster analysis was used to identify tree rings formed under similar climate conditions. Thus, all individual tree rings were classified into 200 classes or clusters of similar climate conditions applied to the rank of the selected precipitation and temperature values using the K-means clustering algorithm as described by Hartigan and Wong (1979). Then, the mean MR frequency and the means of precipitation and temperature were calculated for each cluster. Using this procedure, a robust estimation of the frequency of MR (based on more than 1,000 tree rings for each cluster) was calculated for different ranges of climate conditions. Finally, LRM was calculated using, the mean MR frequencies calculated for each cluster as the dependent variable and the mean temperature, the mean precipitation and their interaction as independent variables.

### Estimation of MR Frequency across the Range Distribution of *Pinus halepensis*

To estimate the predicted frequencies of MR across the distribution range of *P. halepensis*, the previously constructed LMR based with the highest explained variance (*r*^2^) was selected. The selected model was then applied annually from 1902 to 2013 to gridded climatic data across the species distribution area to obtain annually predicted frequencies of MR. Finally, the averages of annual maps were used as a global predicted frequency of MR during the instrumental period (1902–2013).

## Results

### Dendrochronological Network and MR

The extensive dendrochronological network consisted of 113 different sites (Supplementary Table S1) where in total 1,509 trees were selected, ranging from at least form 5 to 35 trees per site. Altogether 2,953 cores were sampled, ranging from at least 6 to 70 samples per site. In total 225,428 tree rings were counted and measured, ranging from at least 330 to 5,363 per site. From the total number of tree rings, 4,150 MR were identified, yielding a global percentage of 1.84. The proportion of MR at sites within the network ranged from 0 to 11.89%.

Cambial age of tree-ring series ranged from 1 to 301 years, with an overall mean of 44 years. Site mean cambial age ranged from 10 to 98 years. Further analysis was conducted on rings with cambial ages from 1 to 169 years to maintain adequate replication. Older rings were represented by fewer than 50 tree ring series and occurred at fewer than 25 of the study sites.

### Age Effect on MR Frequency

**FIGURE 2:**
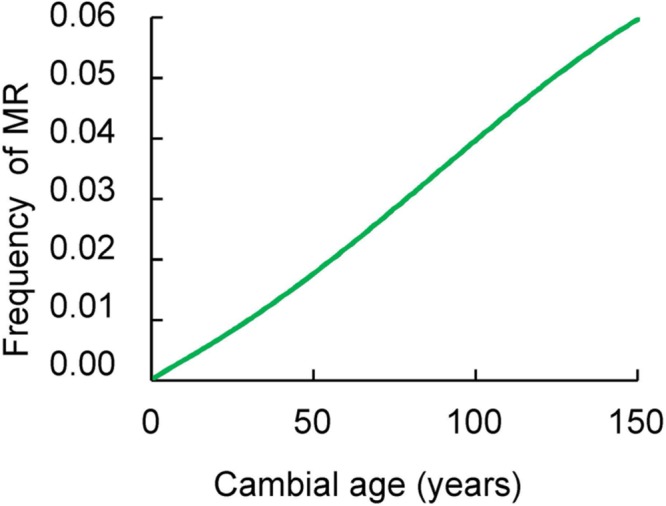
**The relationship between the frequency of MR (vertical axis) and the cambial age (horizontal axis) as described by binomial regression.** Samples were collected at breast height of trees.

The frequency of MR significantly increased with increasing cambial age (**Figure 2**). In tree rings younger than 15 years the proportion of MR was 0.05% with progressive increases until reaching 6.5% at the age of 169 years.

### Observed Frequencies of MR across Dendrochronological Network

**FIGURE 3:**
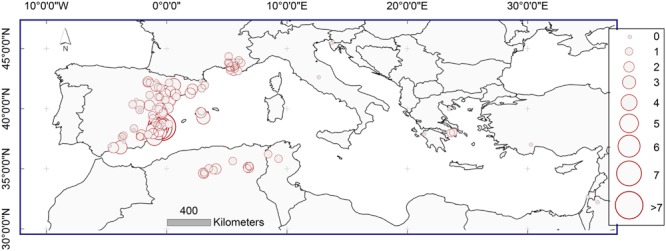
**Mean standardized observed frequency of MR on the study sites represented with red circles.** Meaning of values: 0 – MR were not observed, 1 – observed frequency corresponds to the global average of MR frequency of the whole study area; values 2, 3, 4, 5, 6, 7 indicate the sites where the observed MR frequency was 2, 3, 4, 5, 6, or 7 times higher than the average.

For each sampling site the average standardized observed MR frequency was calculated (**Figure 3**). The value of 0 indicates the sites with no MR; the value of 1 indicates the sites where the observed frequency of MR is equal to the global average of MR frequency of the whole study area. Values of 2, 3, 4, 5, 6, and 7 indicate the sites where the average standardized observed MR frequency is 2, 3, 4, 5, 6 or 7 times higher than the average.

Lower frequencies of MR were found in the northern and eastern portions of the *Pinus halepensis* distribution range in the Mediterranean basin. Higher observed frequencies of MR were located in Spain and northern Africa, with the highest values in south-eastern Spain.

Missing ring frequencies as shown in **Figure 3** are partially comparable among the study sites, because the differences in the frequencies of MR among the populations due to different age structure of populations have been removed. However, a direct comparison may be still biased since obtained frequencies of MR are calculated for different periods at each study site, depending on the length of the available local dataset.

### Climate Conditions Promoting the Occurrence of MR

Goodness-of-fit (*r*^2^) of the 921,600 constructed LRM varies from 0.12 to 0.927 in dependence of selected combination of precipitation and temperature conditions at different time scales.

The highest explained variance of MR occurrence across dendrochronological network (*r*^2^= 0.927) is obtained when model includes the sum of precipitation from previous September to current May, the mean temperature from previous October to current February and their interaction as independent variables.

**FIGURE 4:**
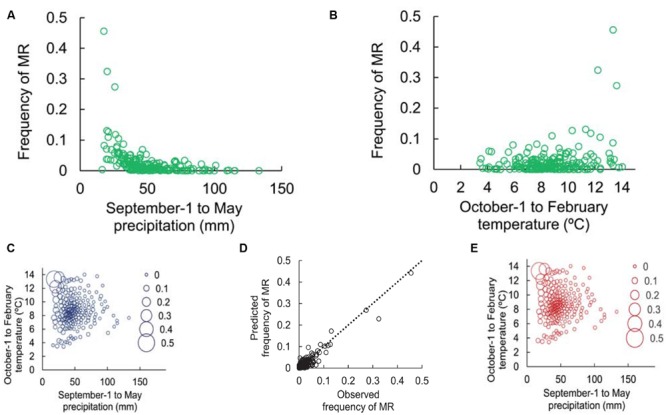
**Observed frequencies of MR calculated for each of 200 clusters of precipitation and temperatures.** Results are based on a model using precipitation from previous September (Sep-1) until current May (8 months period) and temperatures from previous October (Oct-1) until current February (4 months period). Every dot is a cluster, grouping the tree rings from different sites and years from the entire dendrochronological network: **(A)** frequencies of MR vs. mean precipitation of each cluster; **(B)** frequencies of MR vs. mean temperature of each cluster; **(C)** observed frequency of MR for each cluster; **(D)** observed vs. predicted frequency of MR for each cluster; **(E)** predicted frequency of MR for each cluster.

The relationship of MR frequency to climate was non-linear and exponentially increased as the summed total precipitation decreased from the previous September to the current May (**Figure 4A**) and with elevated mean temperature from the previous October to the current February (**Figure 4B**). However, MR was more strongly dependent on the interaction of temperature and precipitation than on either climatic factor taken individually. The frequency of MR for each cluster (**Figure 4C**), the agreement between observed and predicted frequencies of MR (**Figure 4D**), and the predicted frequencies of MR (**Figure 4E**) are shown.

### Estimated Occurrence of MR across the Distribution Range of *Pinus halepensis*

**FIGURE 5:**
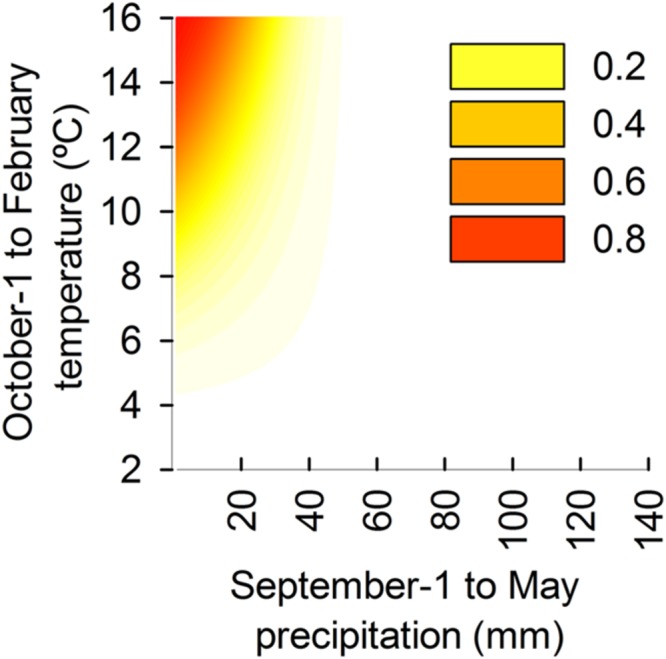
**Estimated frequency of MR predicted from the model based on observed total precipitation from previous September until current May (horizontal axis) and temperatures from previous October until current February (vertical axis).** Climatic signals which determine the occurrence of MR are the combination of high temperatures and low total precipitation. Dark orange color (see the legend) represents the conditions of climate with highest MR frequency (greater than 80%) and clear yellow color represents the conditions of climate with lower MR frequency (less than 20%). White areas indicate that the combinations of temperatures and precipitation are favorable for tree-ring growth and MR are unlikely to occur.

Logistic regression models constructed using summed precipitation from the previous September to current May, mean temperature from the previous October to current February, and their interaction were robust predictors of MR frequency over the wide range of climate conditions and beyond those used to construct the models themselves. Consequently, the model is robust in application for the complete range of climatic conditions across the distribution range of *P. halepensis* (**Figure 5**). The presence of MR is unlikely (frequency lower than 0.05) to occur when total summed precipitation from previous September to current May exceeds 50 mm and when mean temperatures from previous October to current February are lower than 5°C. However, the frequency of MR exponentially increases with concurrent low total precipitation and high temperatures. As a consequence, MR frequency higher that 0.2 can be expected when total summed precipitation are lower than 30 mm coincident with warm temperatures (monthly mean greater than 12°C). MR frequency can be expected to occur in 50% of total tree rings (frequency = 0.5) when precipitation drops below 20 mm and temperatures are greater than 13°C.

Similarly, to have a general view on MR frequency across the species distribution, the selected LRM model was applied annually from the year 1902 until the year 2013 to the gridded climatic data across the distribution area of *P. halepensis*. Predicted frequencies of MR were calculated annually across the species distribution (see complete collection of yearly maps in the Supplementary Material, Supplementary Figure S1). The frequency varied across the distribution area and for particular years, being even higher than 75% in a particular year and site. The highest predicted frequency of MR occurred for south-eastern Spain and northern Algeria. The years of greatest frequency of MR across the species distribution area are also identified (for example 2012, 2005, 1996, 1995, and 1982).

**FIGURE 6:**
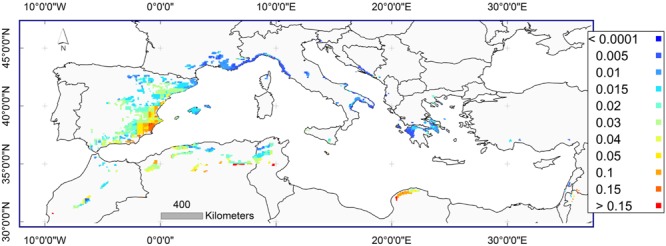
**Average predicted frequencies of MR for the instrumental period 1902–2013 across the distribution area of *Pinus halepensis*.** Dark blue color: frequency below 0.01% (0.0001); dark red color: frequency higher than 15% (0.15).

Yearly calculations of predicted frequencies of MR were averaged for each climatic grid point to represent an unbiased predicted frequency of MR across the distribution range of *P. halepensis* during the instrumental period 1902–2013. The frequency of MR varied across the distribution area with the northern and eastern portion of the range distribution with a predicted frequency of less than 0.01%. The predicted frequency of MR was 15% or higher in the western part of the distribution range in south-eastern Spain and northern Algeria (**Figure 6**). The high predicted values were validated by the high observed frequencies of MR from these locations.

## Discussion

### General Considerations

Dendrochronological dating of tree rings in an annual series is possible due to distinguishable growth increments that can be cross-dated with other series in the sampled population. The crossdated chronologies from this dendrochronological network should provide a reference for future dendrochronological research in the region. By definition, MR cannot be seen and therefore they can confound accurate assignation of calendar dates to rings (e.g., Grundmann et al., 2008). Although not directly countable, the position of MR can be identified through careful crossdating (e.g., Novak et al., 2011). Correctly dated MR can identify the climatic conditions that lead to dormancy of the vascular cambium and the lack of production of an annual ring (Novak et al., 2016). Therefore, MR can be markers for the timing of ECE.

### The Effect of Age on MR

For dendroclimatological analysis of tree-ring width and climate, the age-related trend of MR occurrence needed to be identified and removed prior to determination of the effect of precipitation and temperature on MR. For ring-width series, the most common approach to remove the age- and size-related variation (growth trend) is to fit a curve to the measured ring-width series and then to either subtract or divide the observed measurement from the fitted curve (Cook et al., 1995).

Rather than a quantitative characteristic such as ring width, MR series are binomial since MR are either present or absent for a particular year. Different methods for detrending binomial series in tree rings have been tested so far (e.g., Campelo et al., 2007, 2015; Novak et al., 2013a). Here, we used a recently developed approach which has been applied to intra-annual density fluctuations (Zalloni et al., 2016), and can be used for other binary anatomical characteristics of tree rings.

As trees within the dendroclimatic network increased in age, tree-ring widths tended to narrow and the frequency of MR increased. Ring width can also be related to the length of winter cambial dormancy (e.g., Barnett, 1971) which is largely under genetic control. The genetic potential may become limited by harsh climatic conditions. Ring widths also tend to narrow or be missing in declining trees as in case of silver fir (e.g., Torelli et al., 1986; Bigler et al., 2004). Consequently, the occurrence of MR may indicate tree or forest decline, but rather than being merely a marker of decline, MR formation may also be part of a tree survival strategy. Under extreme conditions, the cambium may produce phloem while producing no xylem (e.g., Gričar et al., 2016; Novak et al., 2016). Annual production of phloem is essential to maintain the distribution pathways of photosynthate and other biomolecules. Although xylem is essential for tree function, the allometric diversion of resources to phloem production likely has survival value under extreme limitations of growth. Therefore, we should consider MR in *P. halepensis* as part of the biological plasticity of the species to adapt to adverse environmental conditions.

### Climate Signals Derived from MR

The relationship between MR and climate is difficult to explore, because the replication of MR across the network studied is low and therefore it is not easy to get robust estimation. The principle of replication represents one of the keys in dendrochronology highlighting the need to use more than one stem radius per tree and more than one tree per site to obtain reliable tree-ring chronologies. Different statistics used to analyze tree-ring series are often based on high number of samples, and normally sampling strategies in dendrochronology are often designed to ensure the requested number of samples.

The next question is how to deal with binomial character of MR, because as a special anatomical feature it cannot be measured but just characterized, based on its presence or absence in a specific year. In this sense, the criteria to define appropriate number of samples to obtain reliable representativeness of MR frequency cannot be based on the same procedure used for tree-ring chronologies. Future investigation and advices how to work with MR are necessary, and a well-designed sampling strategy, would be the best solution.

In this study, to determine the appropriate number of samples to work with MR a novel methodology of global analysis is proposed combining all the data available. It is based on the identifications of analogous climatic conditions which permit joining the tree rings to bigger clusters/classes for which we can get robust estimation of the MR frequency. This method has been applied to intra-annual density fluctuations already (Zalloni et al., 2016).

The disadvantage of our method is that the full value accrues only from dendrochronological networks that span the complete distribution range of the species under investigation. Otherwise the applicability of climatic drivers for MR across the range remains unknown or uncertain.

The advantage of our analysis is the global application to the distribution range of *P. halepensis*. In our case, the global model explained 91.7% of the variability of MR, which stresses that regional rather than local climatic factors are responsible for the occurrence of MR. Periods of extreme drought and warmth were the key factors associated with the occurrence of MR. This finding is supported by previous research on climatic drivers including low moisture availability (Liang et al., 2014) or summer drought (Jonsson et al., 2002; Dulamsuren et al., 2013). The climatic conditions before tree-ring formation can also constrain tree growth (Kuptz et al., 2011; Wettstein et al., 2011; Camarero et al., 2013). In contrast with *P. halepensis* in the Mediterranean Basin, MR in alpine environments were associated with frost events (e.g., Hantemirov et al., 2004; Panayotov et al., 2013).

The climate conditions that triggered the occurrence of MR were mean temperatures higher than 10°C from the previous October through the current February and total accumulated precipitation of less than 50 mm from previous September till current May. Therefore, MR occurred as a consequence of drought before the onset of cambial division and growth. Nine month effect of low precipitation indicates that persistent drought triggers MR. The same occur also with high temperatures but on even shorter temporal scale during 5 months before the beginning of growth. High temperatures and prolonged drought may exhaust the energy reserves necessary for growth.

Importantly, the combination of high temperatures and low precipitation was identified as the trigger for MR. The identification of this combined role was shown for *P. halepensis* by de Luis et al. (2013) and for other tree species (Esper et al., 2007b; Rammig et al., 2015; Seim et al., 2015).

Normally, the climate thresholds are not being explored in dendrochronology. They are, however, essential to evaluate the importance of extremes, (i.e., ECE) on tree growth, and to define the range of climatic conditions associated to the occurrence of MR. The thresholds that trigger MR, therefore may be used to define ECE for the Mediterranean Basin.

Under favorable growing conditions, the vascular cambium of *P. halepensis* may be active throughout the entire year. For most growing seasons, cell division ceases during the winter due to low temperatures and/or dry conditions, resuming growth with increased availability of moisture (Liphschitz et al., 1984; Liphschitz and Lev-Yadun, 1986; de Luis et al., 2011a). Under extreme limiting conditions, the vascular cambium does not produce new cells, and a MR occurs in the tree-ring series (Novak et al., 2016). As we know so far, this is the first time that MR (which can be interpreted as a consequence of extreme growth events) are related to ECE. We demonstrated that the relationship of MR to climate was not linear. We also presented LRM as a method for analysis.

Moreover, MR seem to be promising tree-ring features linked to the occurrence of extreme events of climate, but also to adverse growth conditions, as the competition for light in different species (e.g, Lorimer et al., 1999; Parent et al., 2002). In some cases more than 50 tree rings can be omitted during suppressed a period, which reduces usefulness of these samples-species for dendrochronological studies. MR due to competition for light are mainly linked to the young phase of shade tolerant species, while in the case of *Pinus halepensis* they are not in sequences but erratically driven by climatic and so increase with tree age. For this reason an analysis of synchronization of MR will be very informative because it can be used to separate ECE form other extreme environmental events, like volcanic eruptions (D’Arrigo et al., 2013) or severe frost or drought from suppression or dieback phenomena. In the latter case MR are very close one to the other and in sequences within small tree-rings.

### Distribution of MR

Spatial and temporal distribution of MR frequencies showed differences across the distribution area *of P. halepensis* in the Mediterranean. MR were more frequent in the western portion of the distribution range including south-eastern Spain and northern Algeria, and less frequent in the northern and eastern portion of the range such as northern Spain, France, central Italy, Slovenia, and along the coast of Greece and Croatia.

Current predictions of climate change (IPCC, 2013) include a greater increase of winter than summer temperatures and a general decrease of precipitation, both of which are likely to increase the occurrence of MR. MR are relatively frequent in south-eastern Spain but absent from the northern part of distribution of the species. Our findings are supported by St. George et al. (2013) who found that for trees in the Northern Hemisphere (which also includes Mediterranean), MR are most common in trees at sites where growth is limited by moisture availability. As climate continues to change, the relationship of MR to climate may also change. The open question remains as to whether the increasing occurrence of MR in recent years should be attributed to natural climate variability or to global climate change (Allen et al., 2010).

## Conclusion

The presence of MR is significantly related to the tree-ring age. The result showed an increase of MR frequency with increasing cambial age.

Across the distribution range of *P. halepensis*, MR formation was triggered by the combination of high mean temperatures from previous October till current February and scarce accumulated precipitation from previous September till current May. This is a global conclusion and can be applied to every site across the distribution range.

This research is the first to identify spatial and temporal variation of the frequency of MR in an extensive dendrochronological network. Our method allows extending the results to the entire range of *P. halepensis* in the Mediterranean Basin. Identification of the frequency and position of MR should facilitate future crossdating and construction of tree-ring chronologies. The occurrence of MR can identify ECE of the past and support predictions of the effects of climate change for the future.

## Author Contributions

Conception and design of the study were performed by KN and MD; acquisition of data was realized by KN, MD, MS, LL, RS-N, KČ, JG, AD, GP, CR, and AP; analysis of data was performed by KN, MD, JR, KS, CR; interpretation of data was realized by KN, MD, KČ, JG, AD, GP, CR, and KS; drafting and writing the work was performed by KN, MD, MS, LL, RS-N, and KČ; critical revision of work was performed by KN, MD, JR, KČ, JG, AD, GP, CR, AP, and KS. All the authors discussed and commented on the manuscript, gave final approval to be published, and agreed on the integrity of the work.

## Conflict of Interest Statement

The authors declare that the research was conducted in the absence of any commercial or financial relationships that could be construed as a potential conflict of interest.

## Footnotes

1 www.rinntech.com
