# Supplementary material for: Missing Rings in Pinus halepensis – The Missing Link to Relate the Tree-Ring Record to Extreme Climatic Events
Source: Front Plant Sci. 2016 May 31;7:727. doi: 10.3389/fpls.2016.00727 (PMC4885872; doi:10.3389/fpls.2016.00727)
Supplement: Supplementary file 2 [file Data_Sheet_1.DOCX]

Supplementary Material

**Missing rings in *Pinus halepensis* – the missing link to relate the tree-ring record to extreme climatic events**

K. Novak^1,2*^, M. De Luis^1^, M.A. Saz^1^, L.A. Longares^1^, R. Serrano-Notivoli^1^, J. Raventós^2^, K. Čufar^3^, J. Gričar^4^, A. Di Filippo^5^, G. Piovesan^5^, C.B.K. Rathgeber^6^, A. Papadopoulos^7^, K.T. Smith^8^

*** Correspondence:** Corresponding Author: [knovak@unizar.es](mailto:knovak@unizar.es)

# Supplementary Tables and Figures

**Supplementary Table 1 (Table S1).** Description of 113 sampling sites: order, country, site, site code, elevation, latitude, longitude, number of trees, number of cores, number of tree rings, chronology time span, length, number of MR per site, frequency of MR per site.

**Supplementary Figure** **1 (Figures S6).** Annual predicted frequencies of MR across the distribution area of *Pinus halepensis* in the Mediterranean Basin for every year from 1902 till 2013. Dark blue colour: the frequency is below 0.1% (0.001); dark red colour: the frequency is higher than 75% (0.75).
