# Supplementary material for: Missing Rings in Pinus halepensis – The Missing Link to Relate the Tree-Ring Record to Extreme Climatic Events
Source: Front Plant Sci. 2016 May 31;7:727. doi: 10.3389/fpls.2016.00727 (PMC4885872; doi:10.3389/fpls.2016.00727)
Supplement: Supplementary file 3 [file Image_1.PDF]

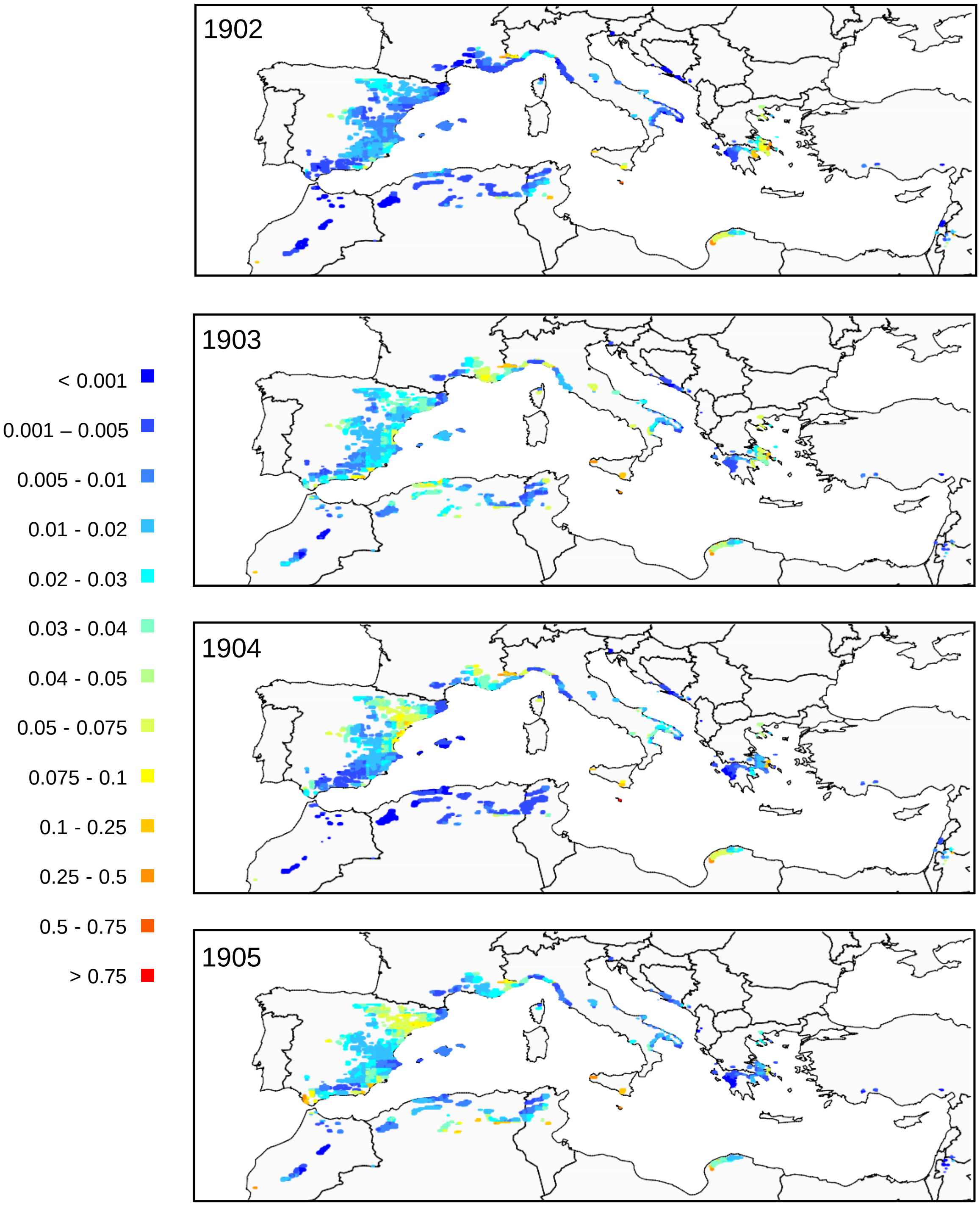

Predicted frequencies of MR across the distribution area of *Pinus halepensis* in the Mediterranean Basin (1902-1905).

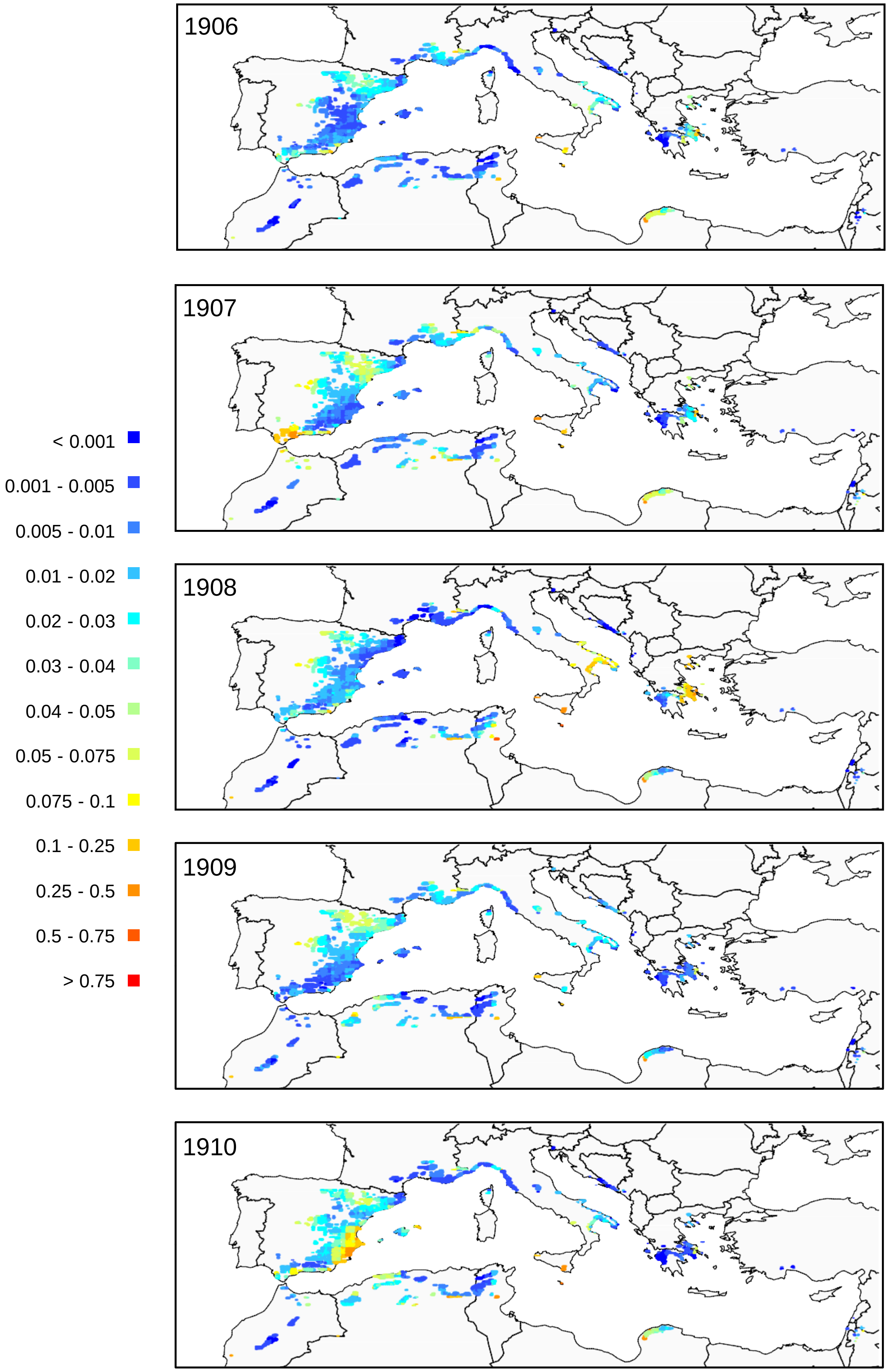

Predicted frequencies of MR across the distribution area *of Pinus halepensis* in the Mediterranean Basin (1906-1910).

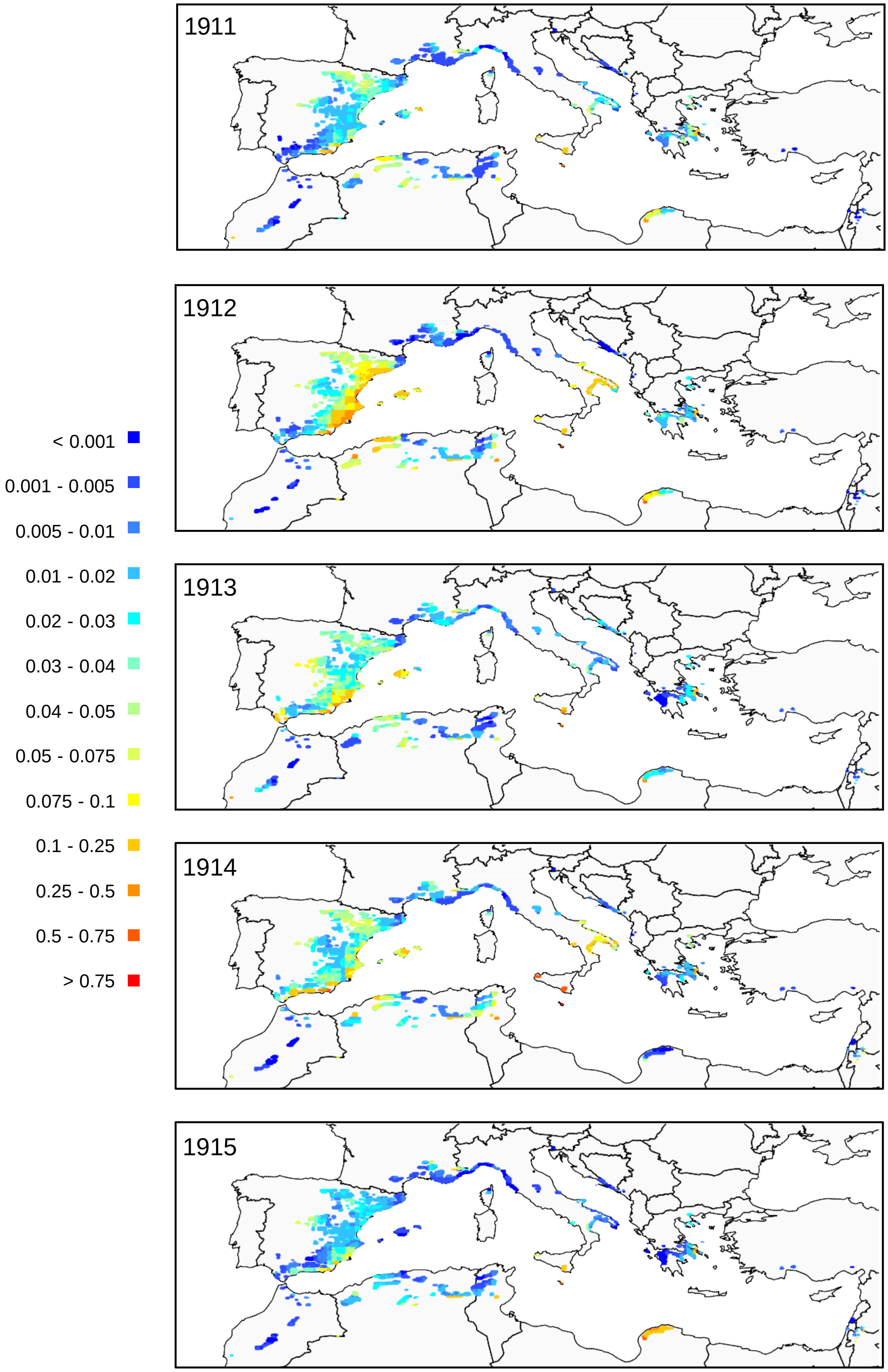

Predicted frequencies of MR across the distribution area of *Pinus halepensis* in the Mediterranean Basin (1911-1915).

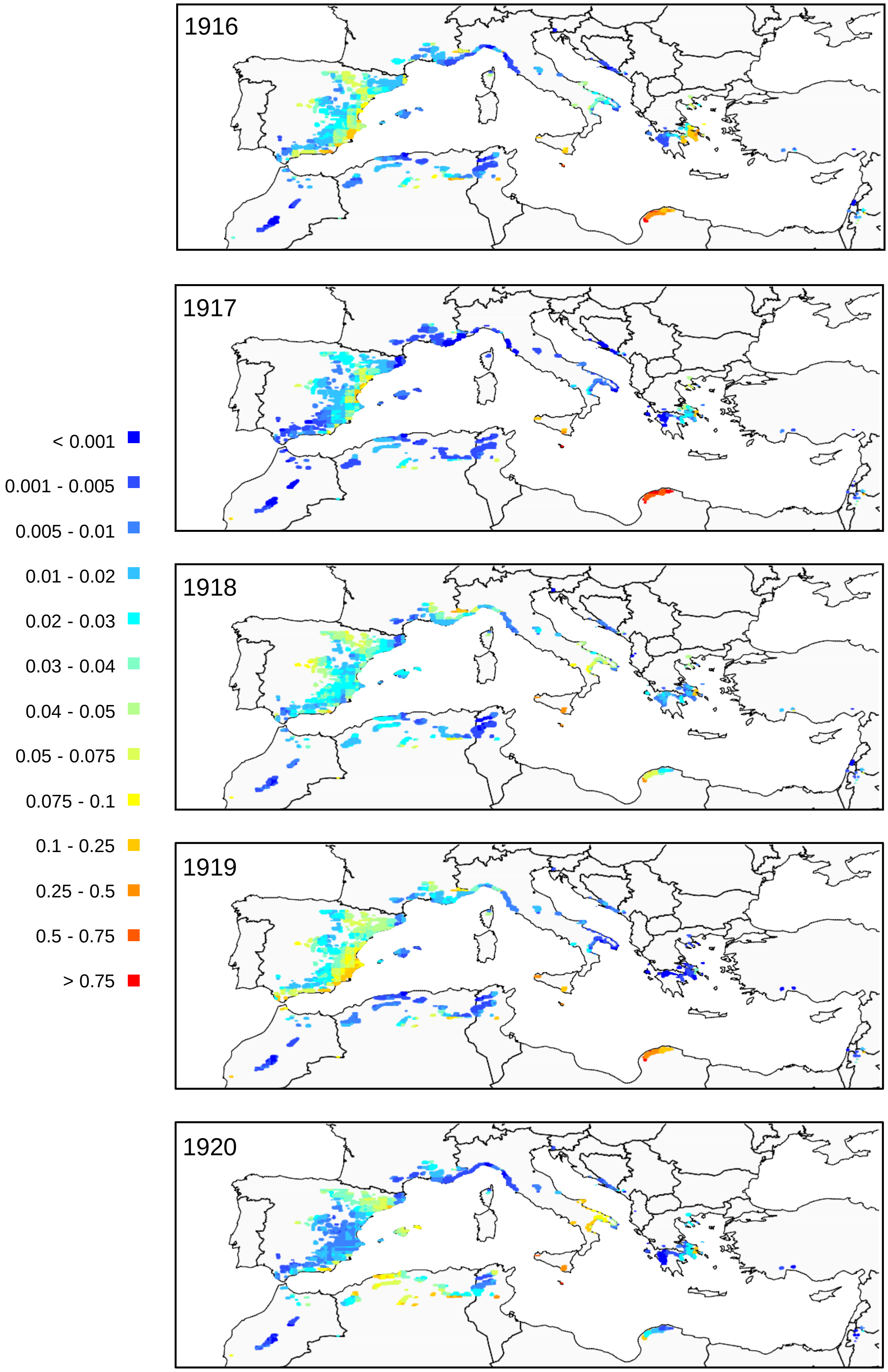

Predicted frequencies of MR across the distribution area of *Pinus halepensis* in the Mediterranean Basin (1916-1920).

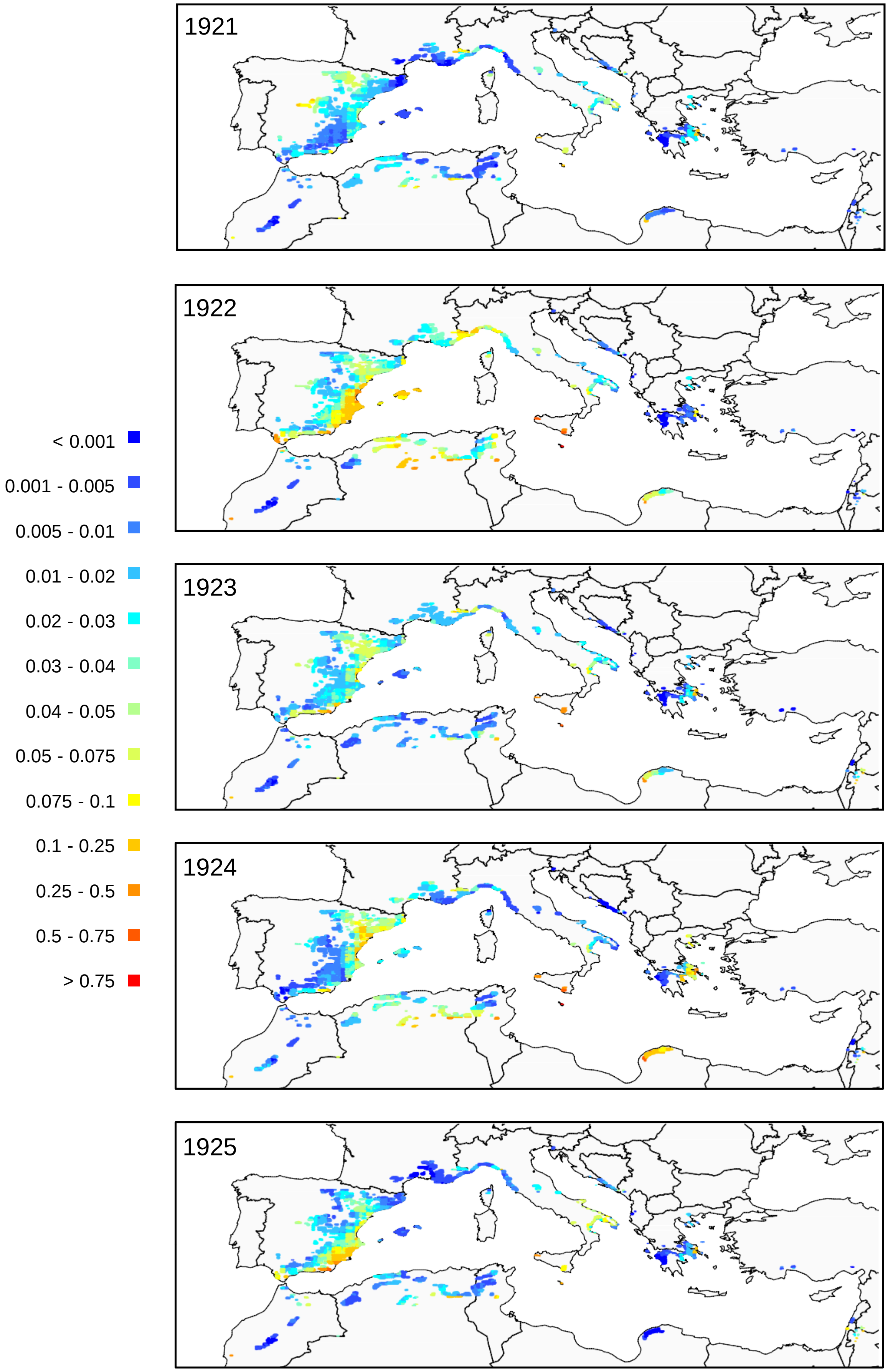

Predicted frequencies of MR across the distribution area of *Pinus halepensis* in the Mediterranean Basin (1921-1925).

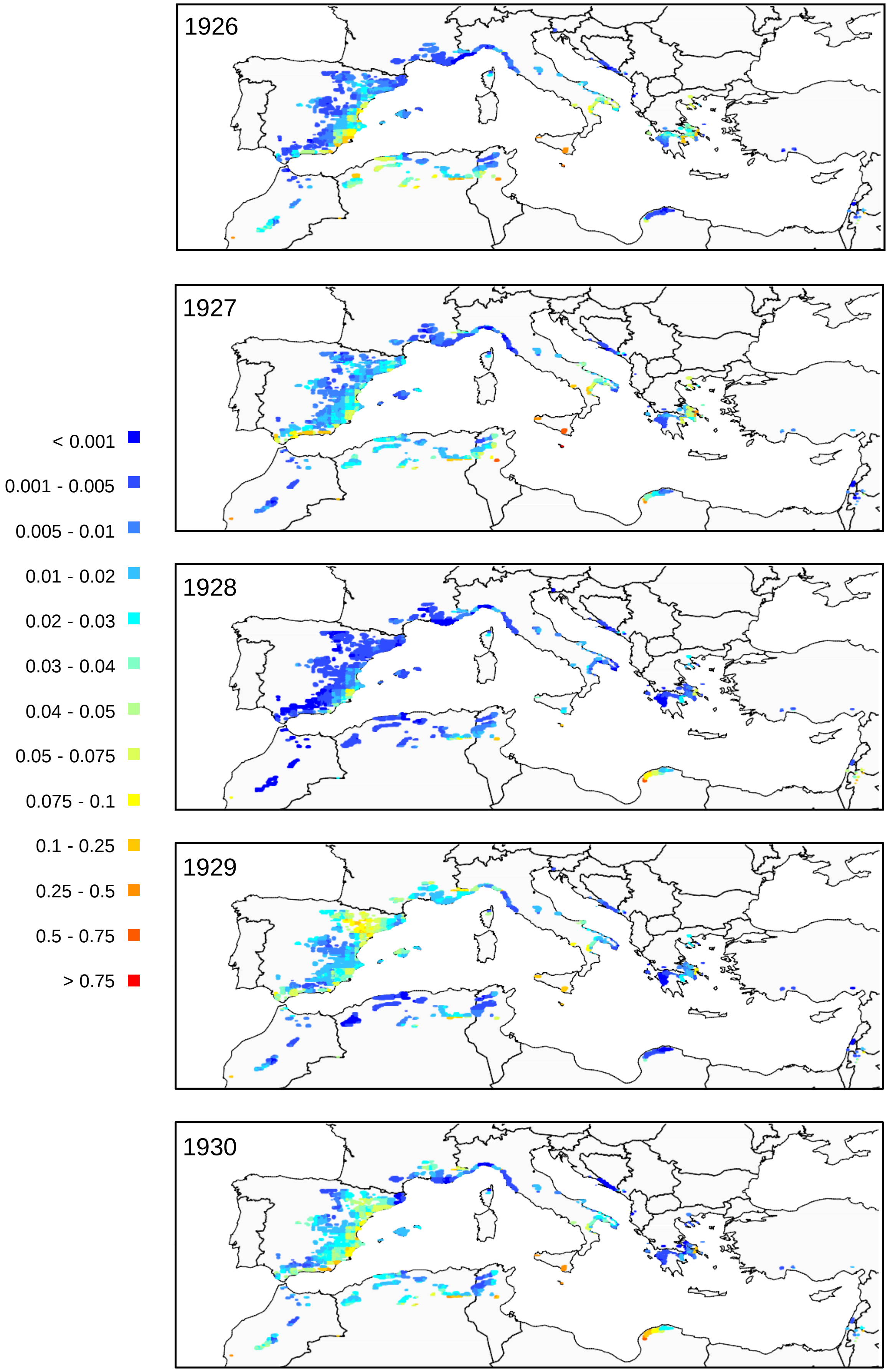

Predicted frequencies of MR across the distribution area *of Pinus halepensis* in the Mediterranean Basin (1926-1930).

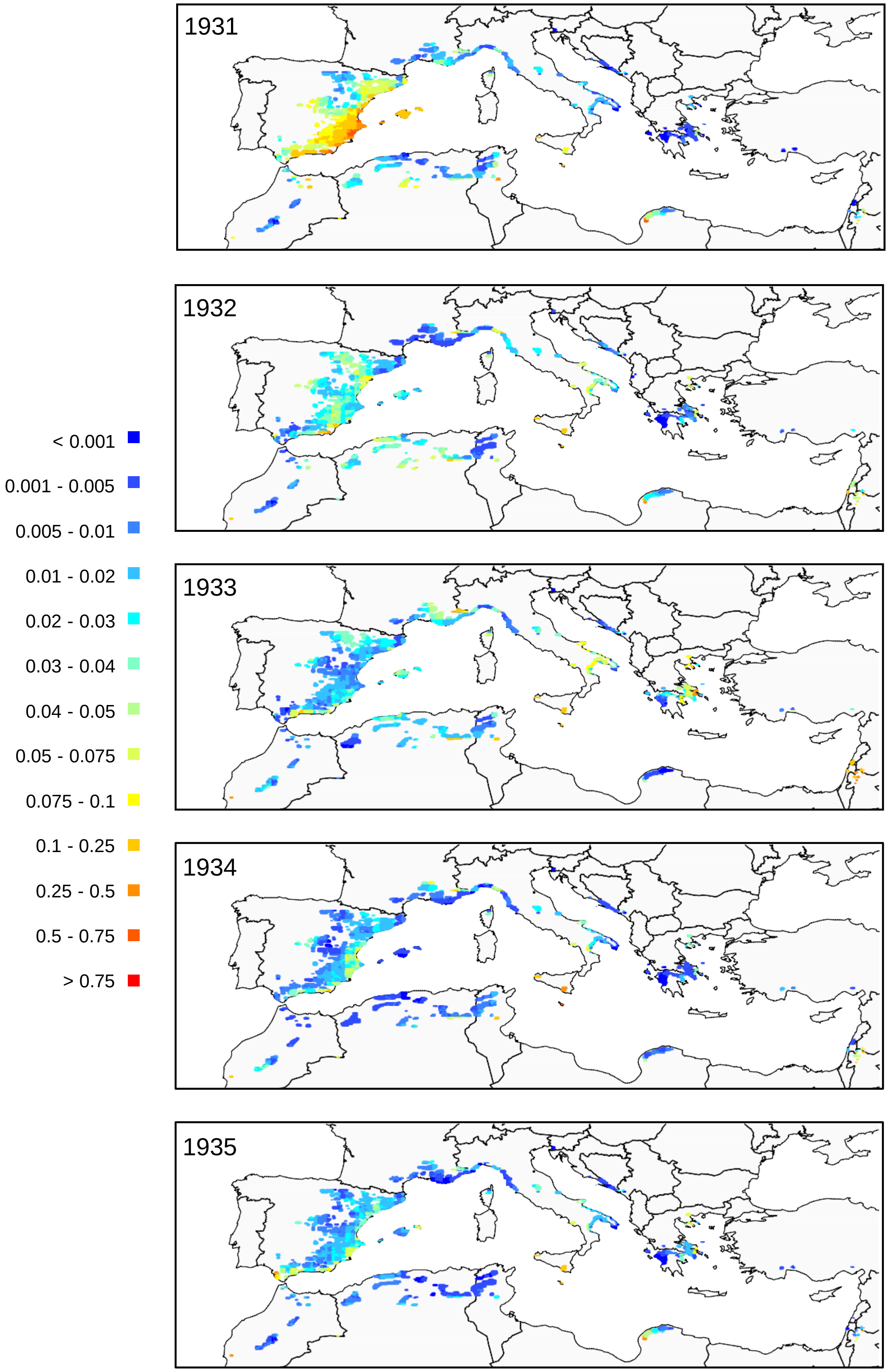

Predicted frequencies of MR across the distribution area of *Pinus halepensis* in the Mediterranean Basin (1931-1935).

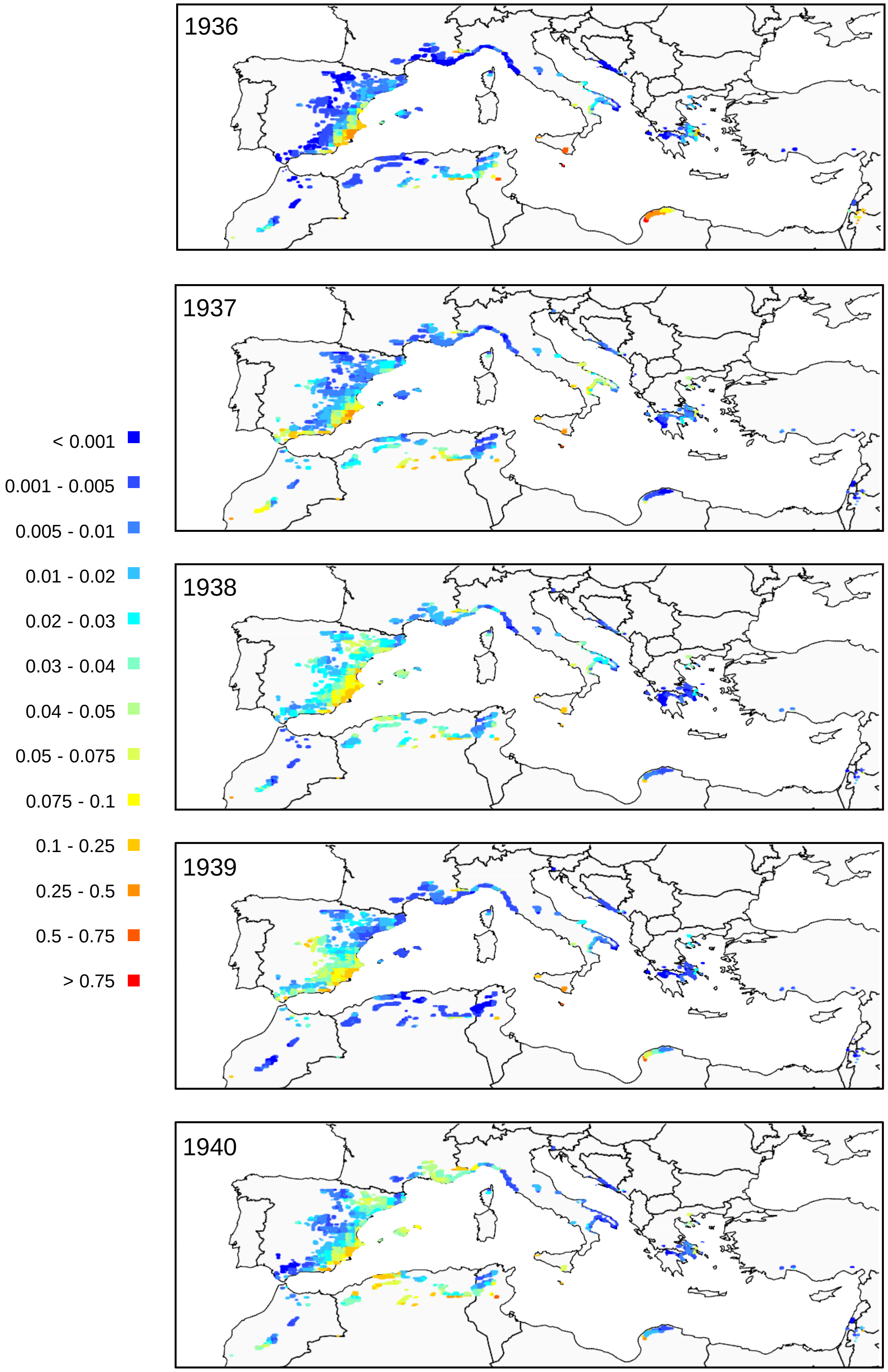

Predicted frequencies of MR across the distribution area of *Pinus halepensis* in the Mediterranean Basin (1936-1940).

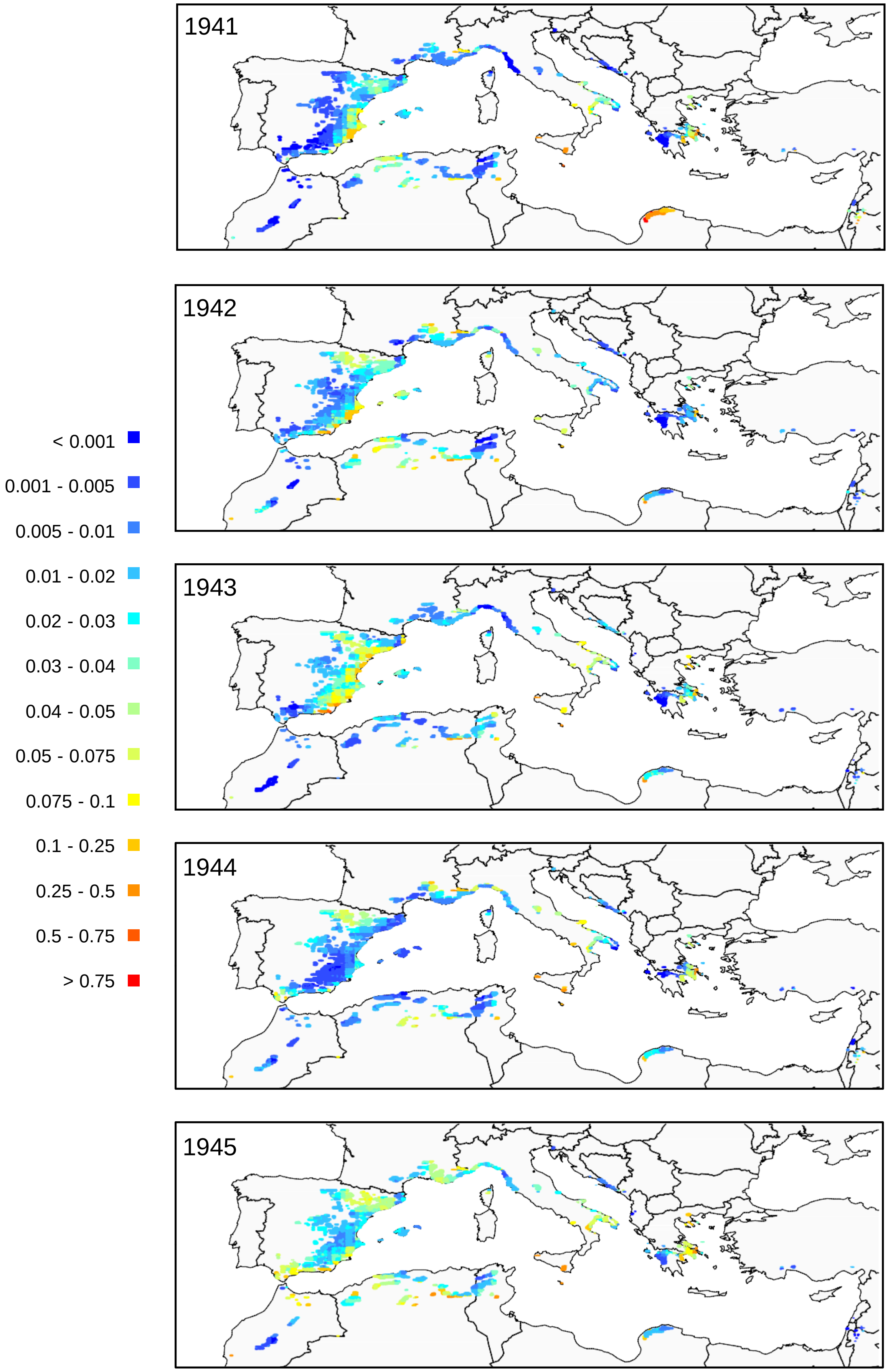

Predicted frequencies of MR across the distribution area of *Pinus halepensis* in the Mediterranean Basin (1941-1945).

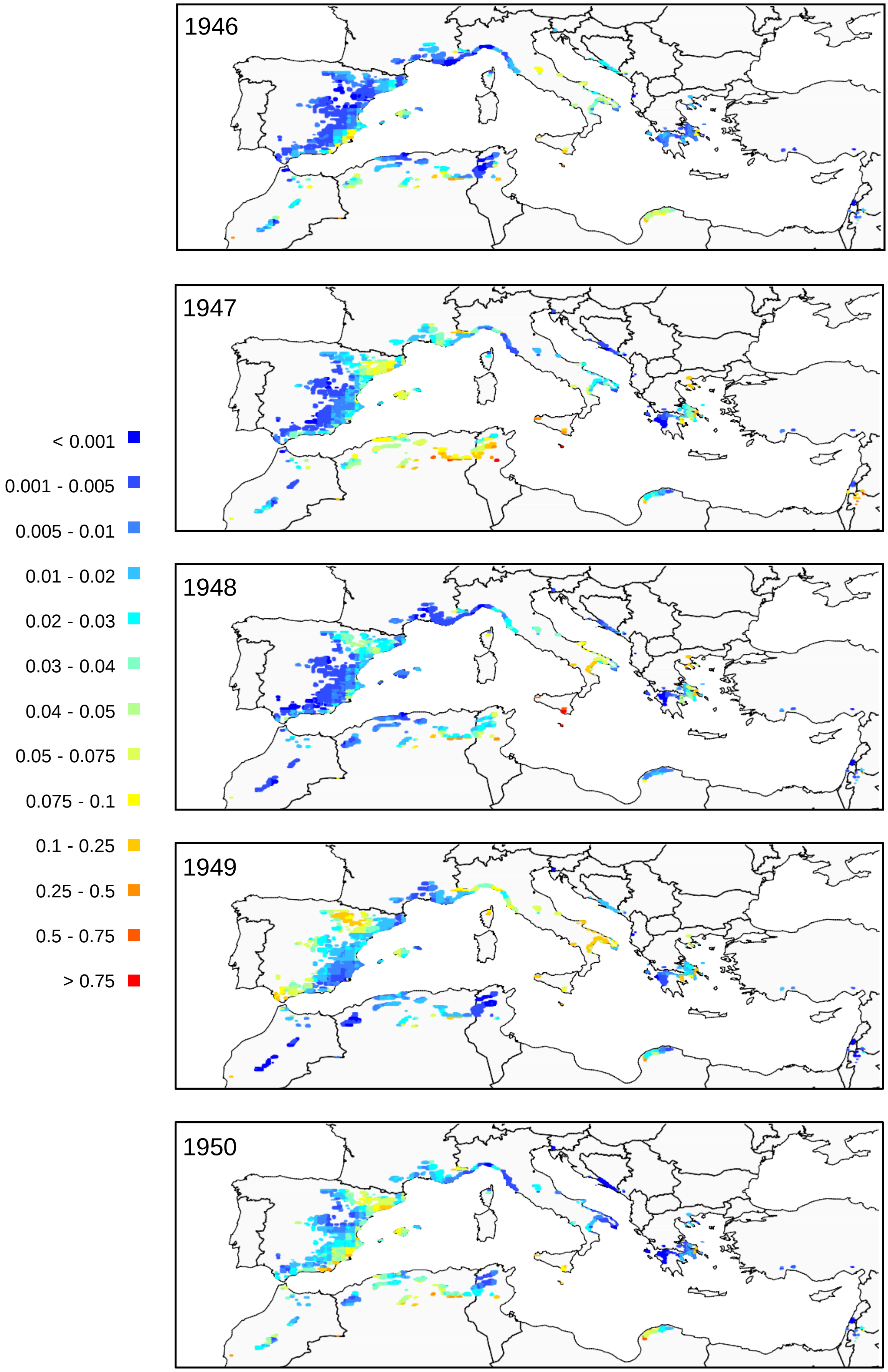

Predicted frequencies of MR across the distribution area of *Pinus halepensis* in the Mediterranean Basin (1946-1950).

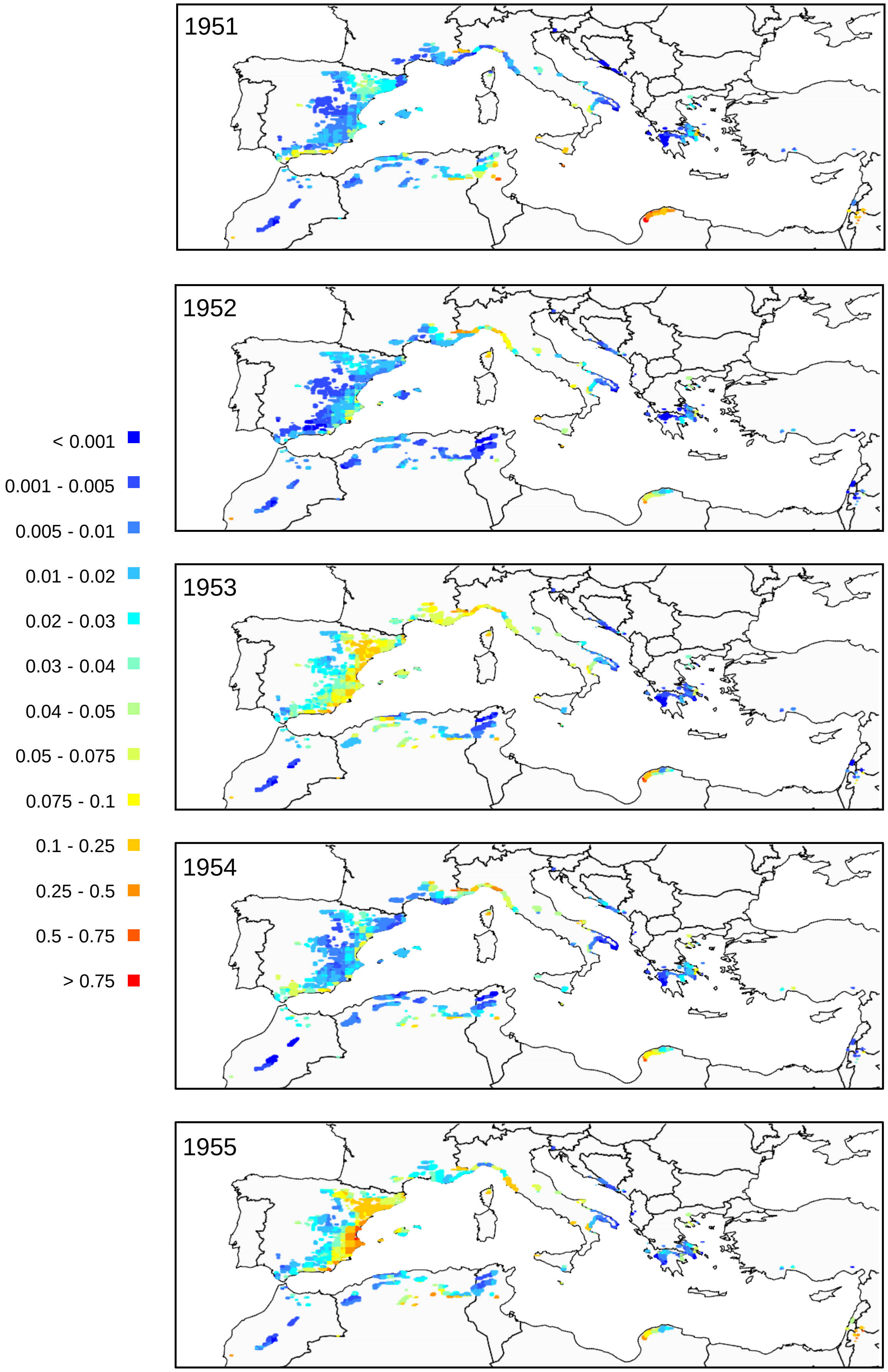

Predicted frequencies of MR across the distribution area of *Pinus halepensis* in the Mediterranean Basin (1951-1955).

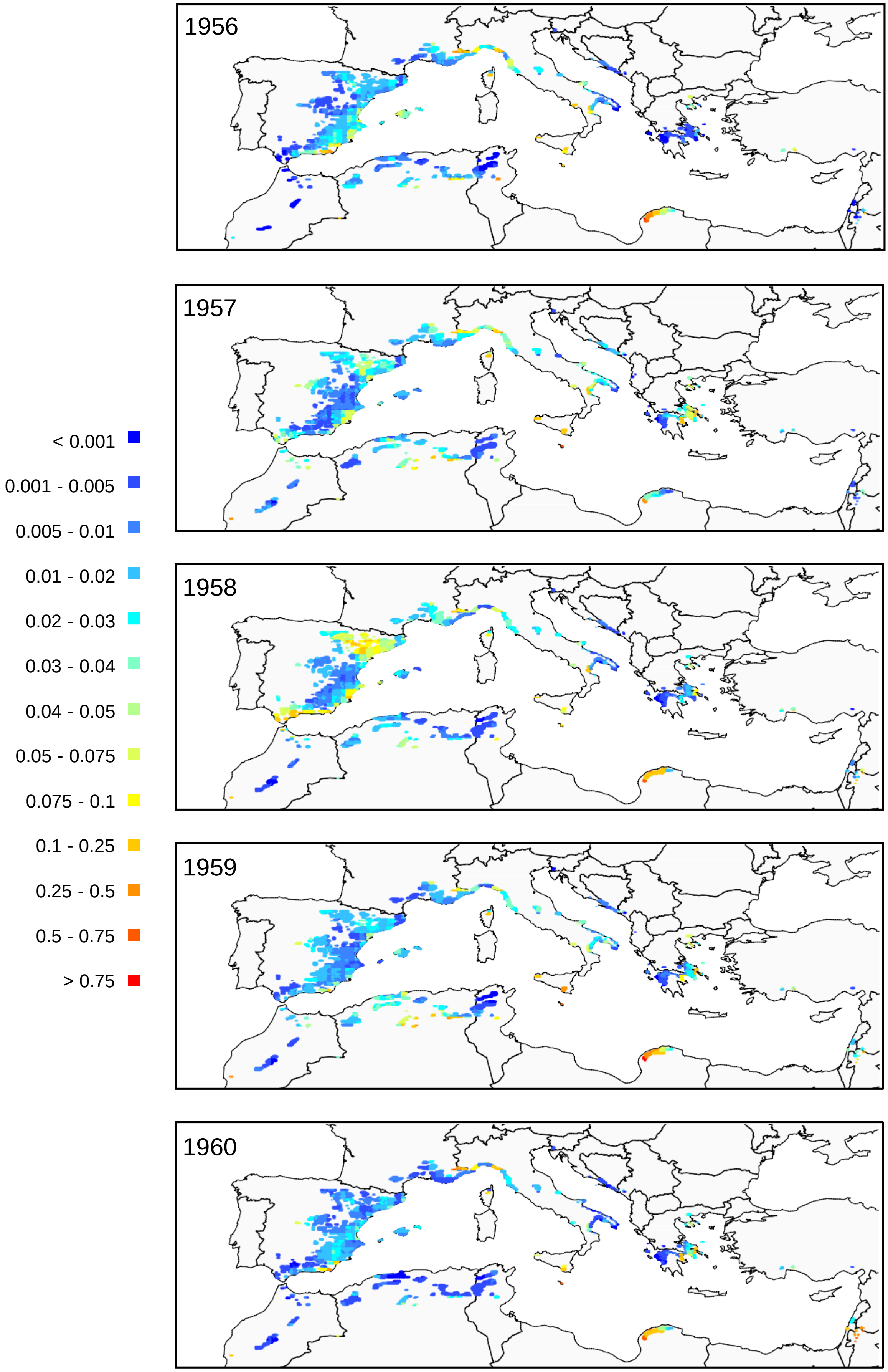

Predicted frequencies of MR across the distribution area of *Pinus halepensis* in the Mediterranean Basin (1956-1960).

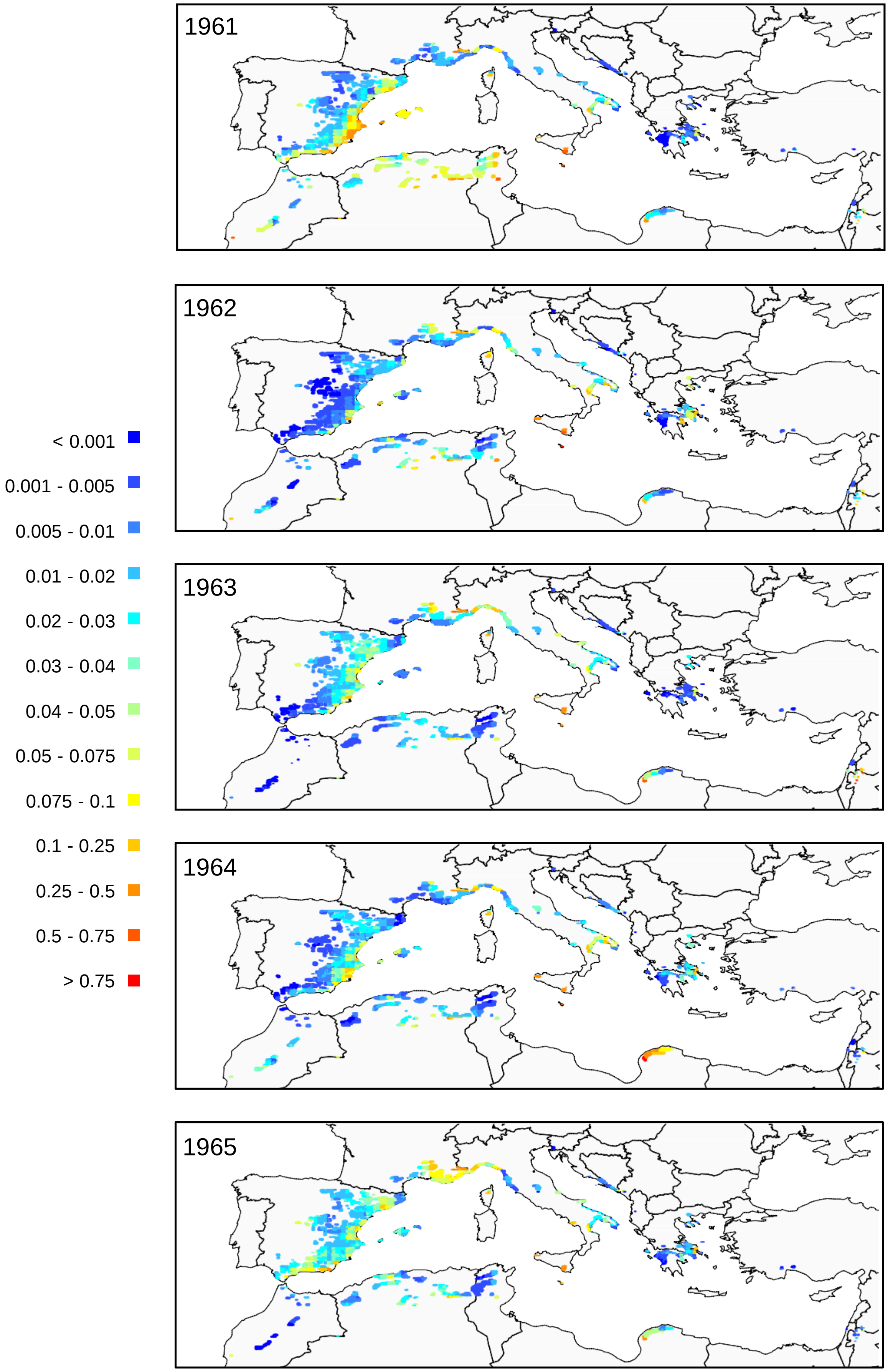

Predicted frequencies of MR across the distribution area of *Pinus halepensis* in the Mediterranean Basin (1961-1965).

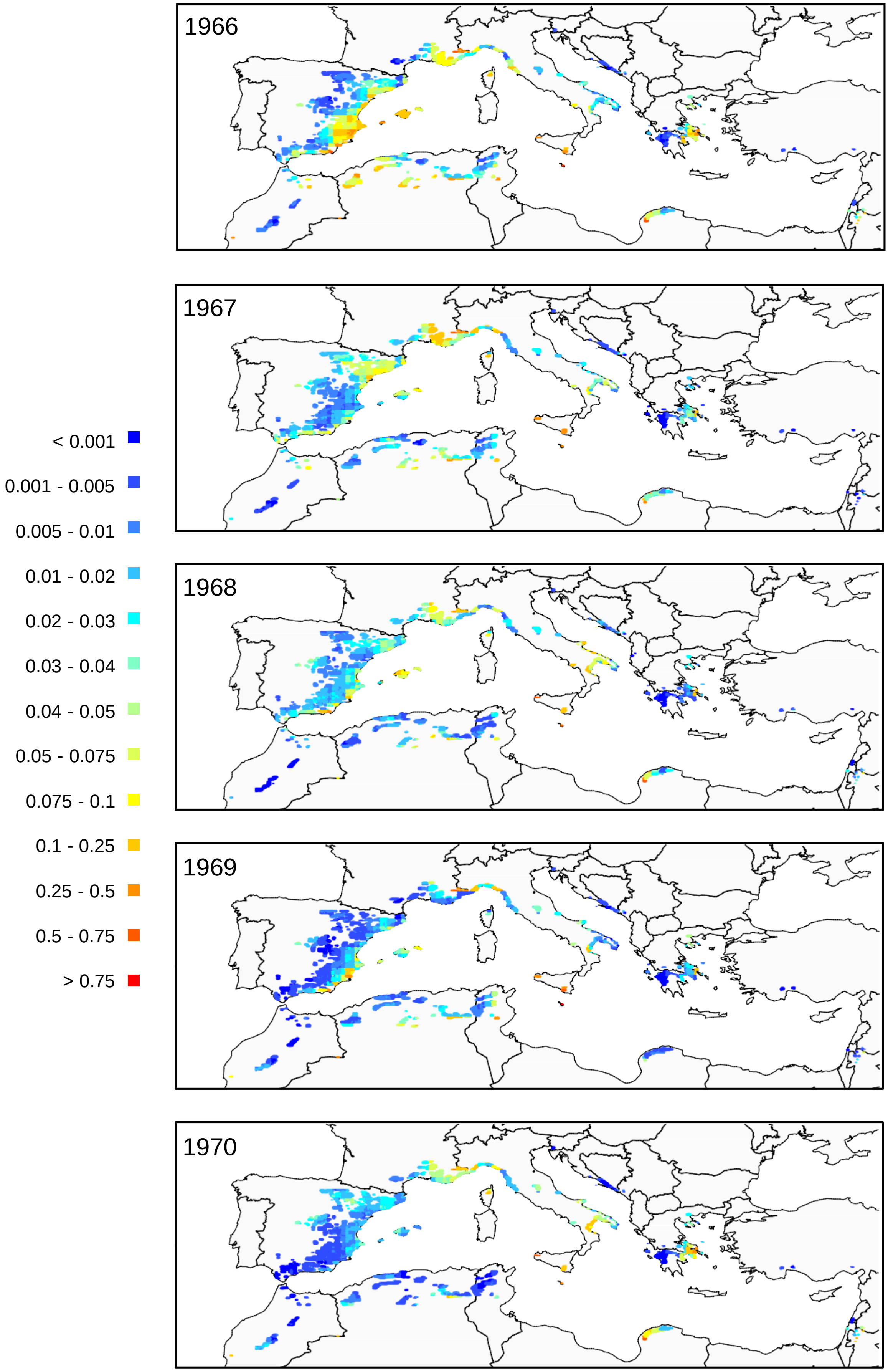

Predicted frequencies of MR across the distribution area of *Pinus halepensis* in the Mediterranean Basin (1966-1970).

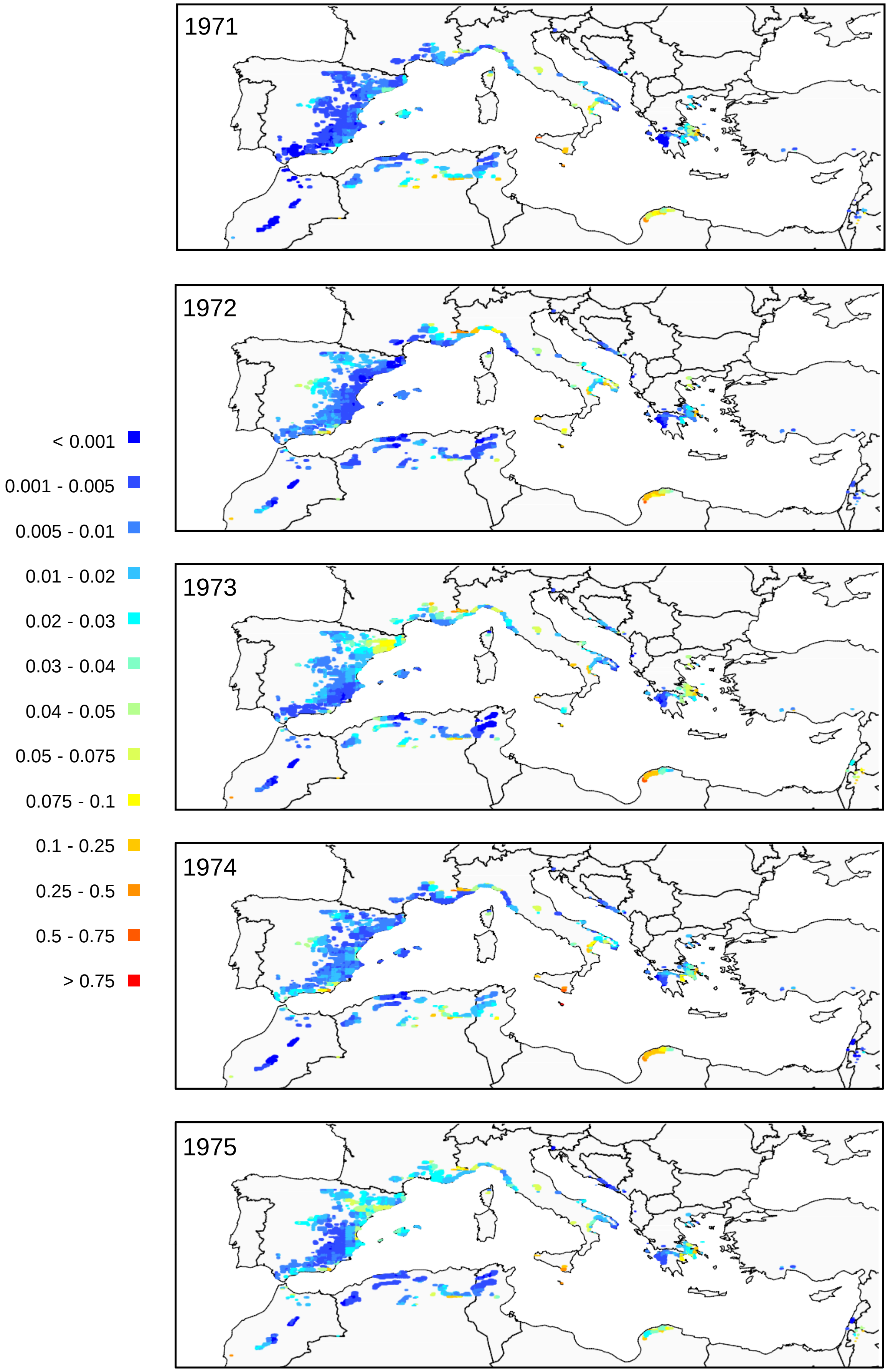

Predicted frequencies of MR across the distribution area of *Pinus halepensis* in the Mediterranean Basin (1971-1975).

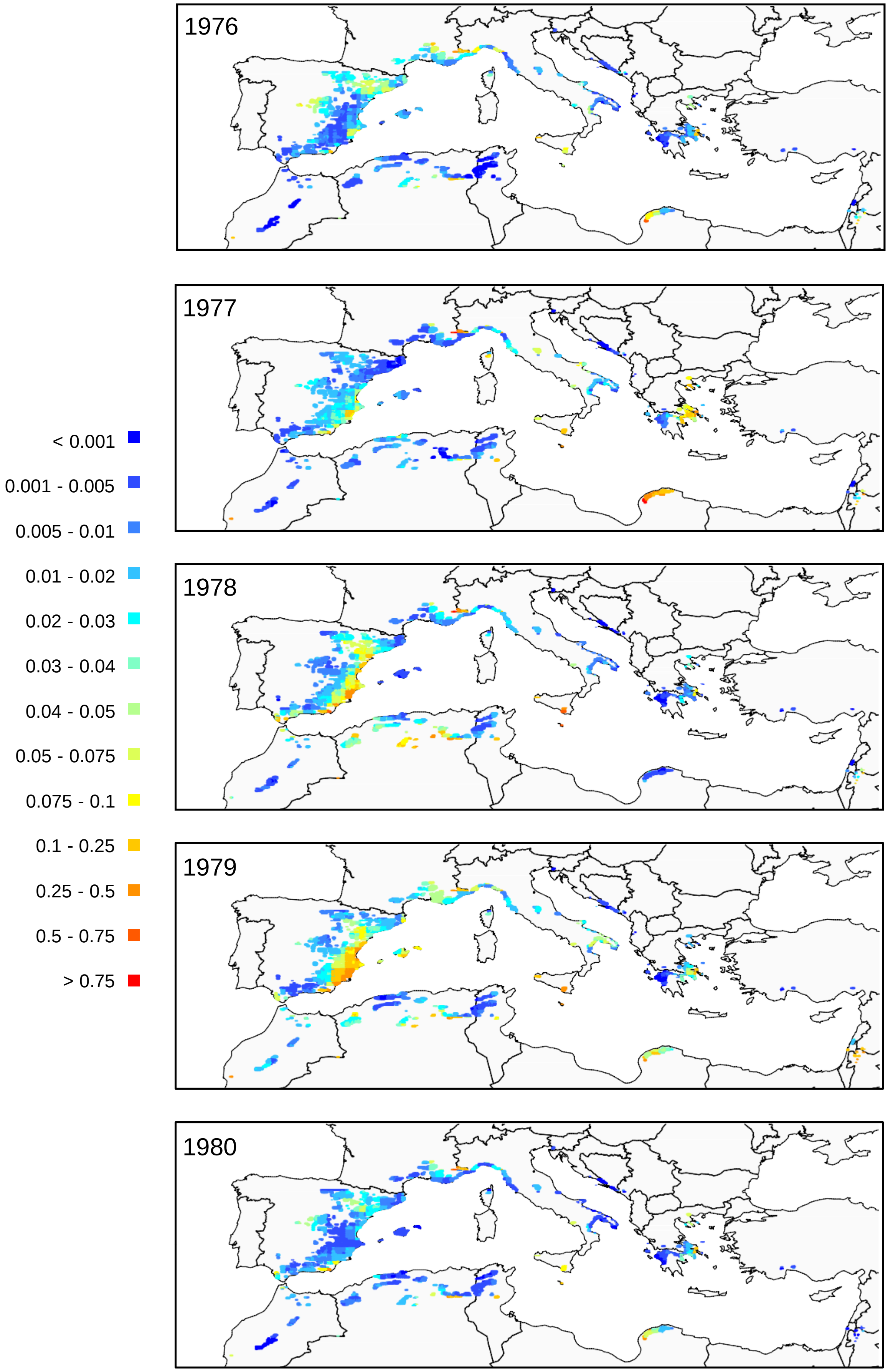

Predicted frequencies of MR across the distribution area of *Pinus halepensis* in the Mediterranean Basin (1976-1980).

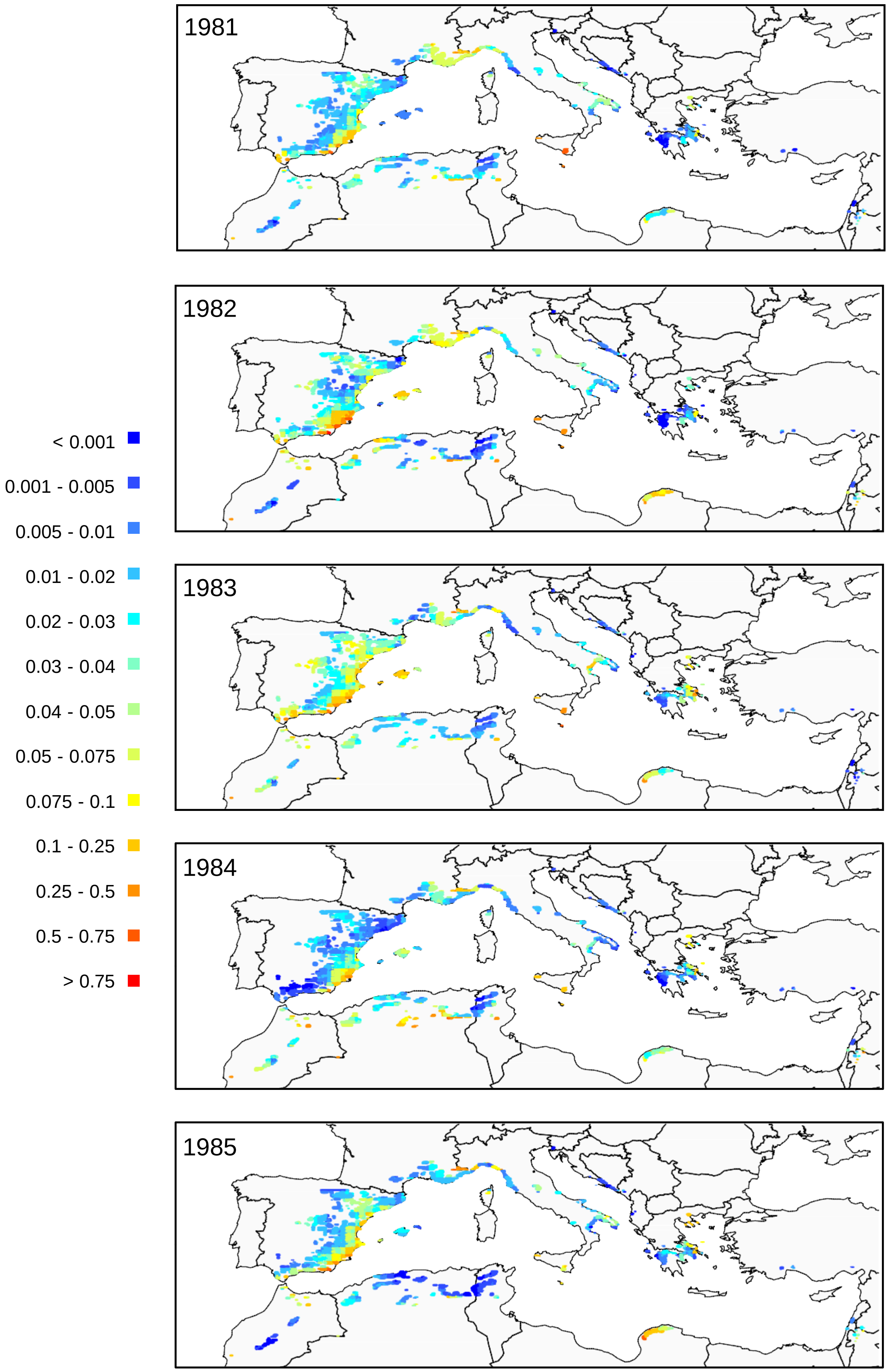

Predicted frequencies of MR across the distribution area of *Pinus halepensis* in the Mediterranean Basin (1981-1985).

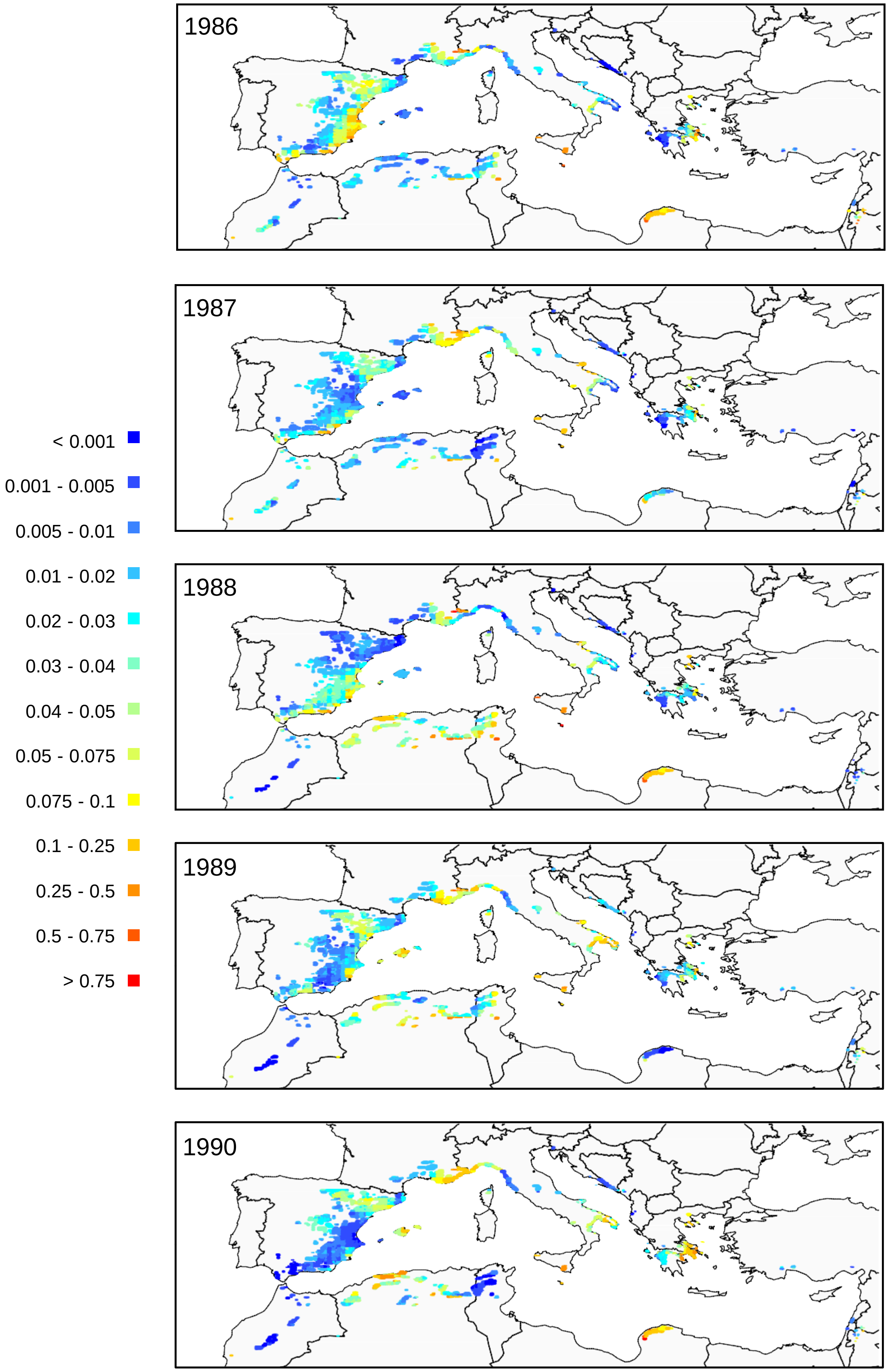

Predicted frequencies of MR across the distribution area of *Pinus halepensis* in the Mediterranean Basin (1986-1990).

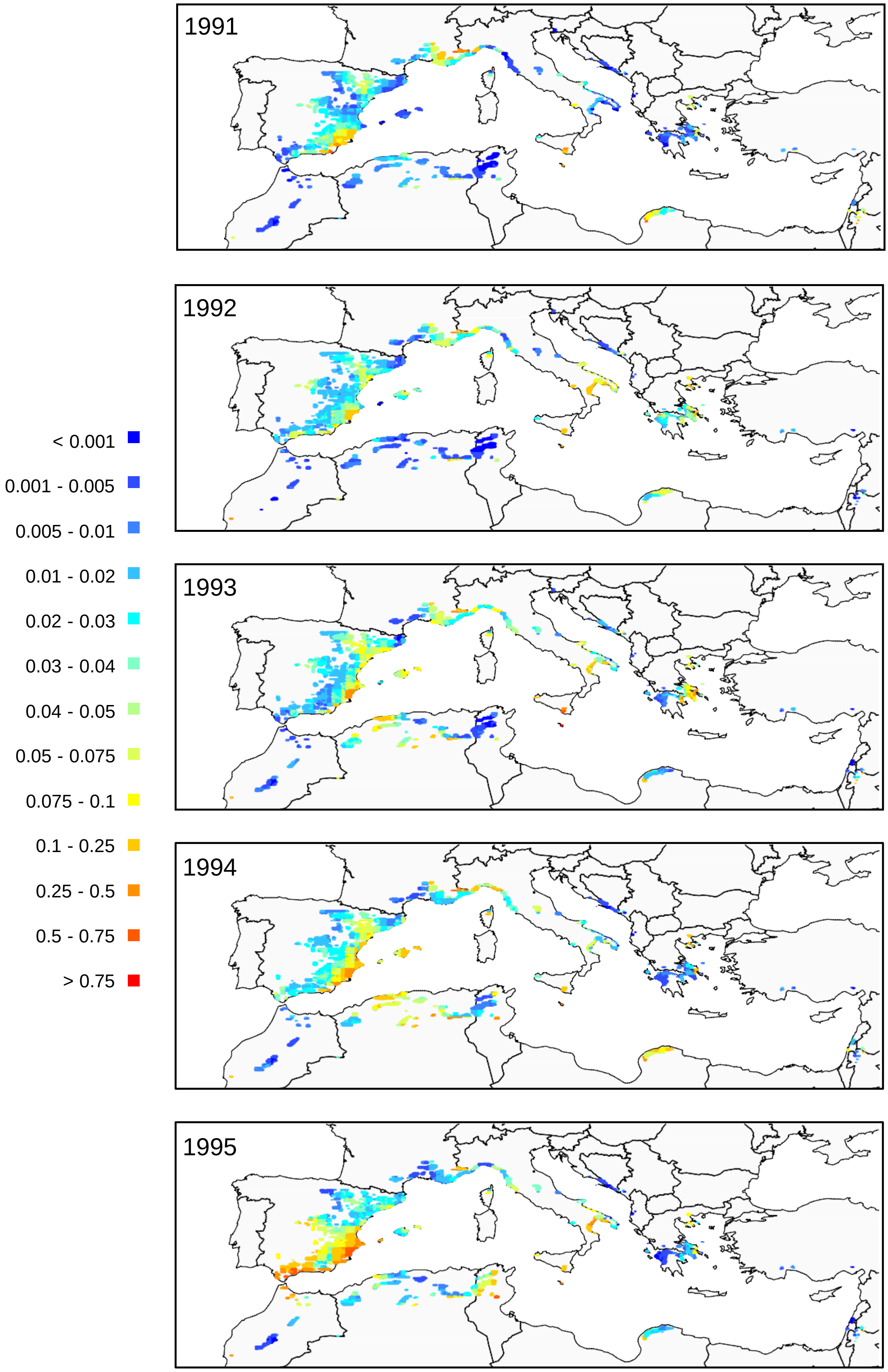

Predicted frequencies of MR across the distribution area of *Pinus halepensis* in the Mediterranean Basin (1991-1995).

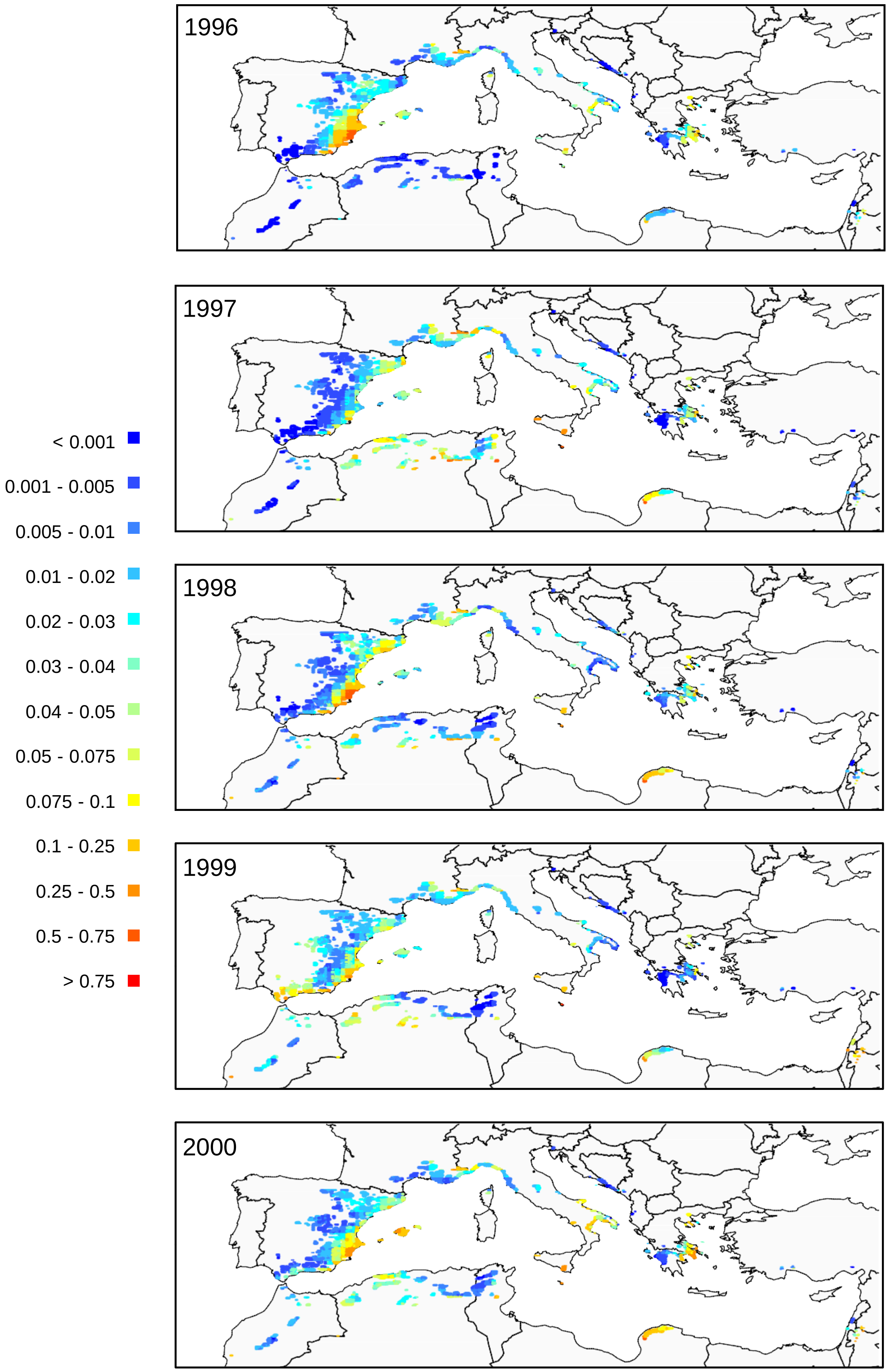

Predicted frequencies of MR across the distribution area of *Pinus halepensis* in the Mediterranean Basin (1996-2000).

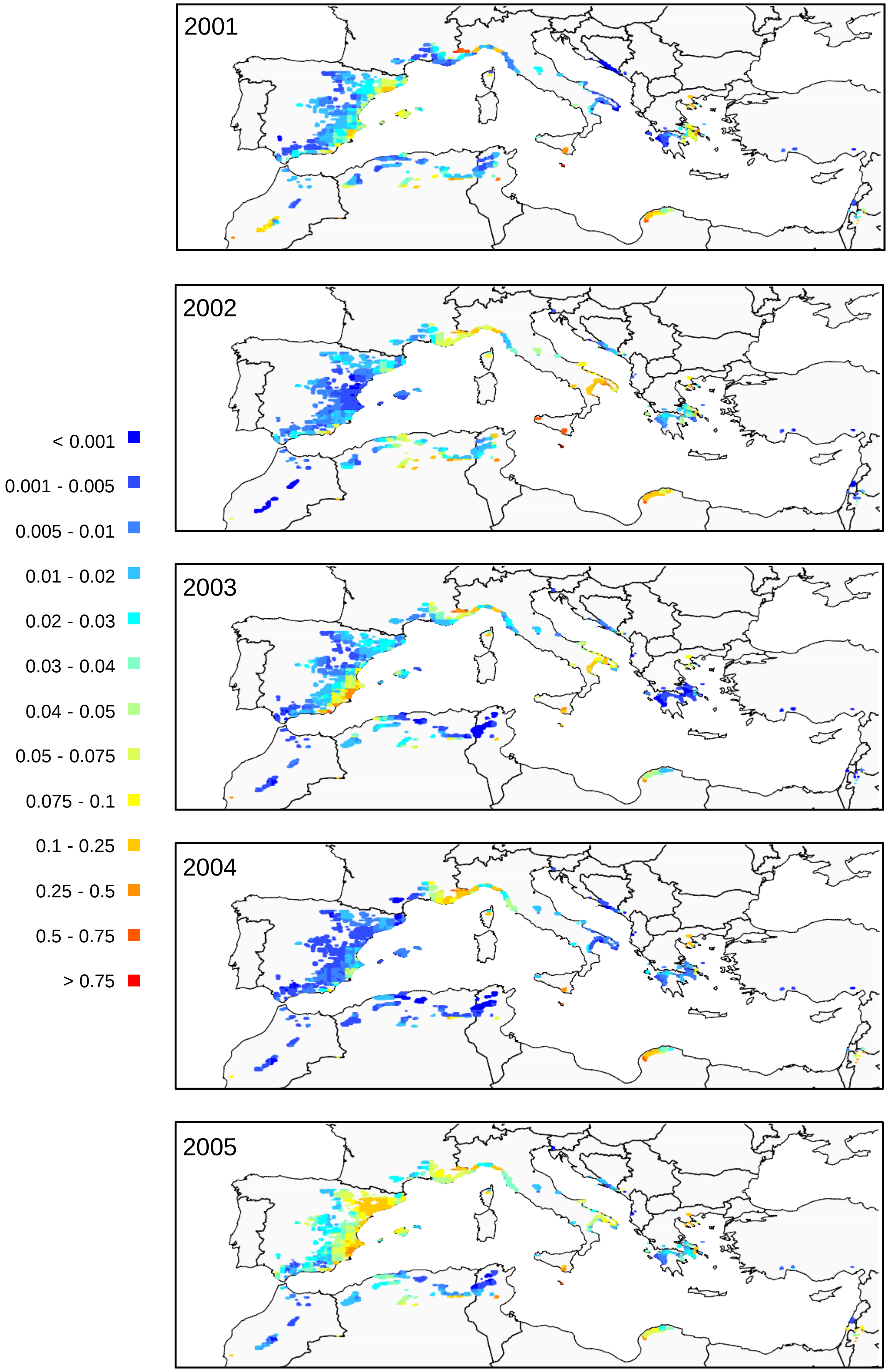

Predicted frequencies of MR across the distribution area of *Pinus halepensis* in the Mediterranean Basin (2001-2005).

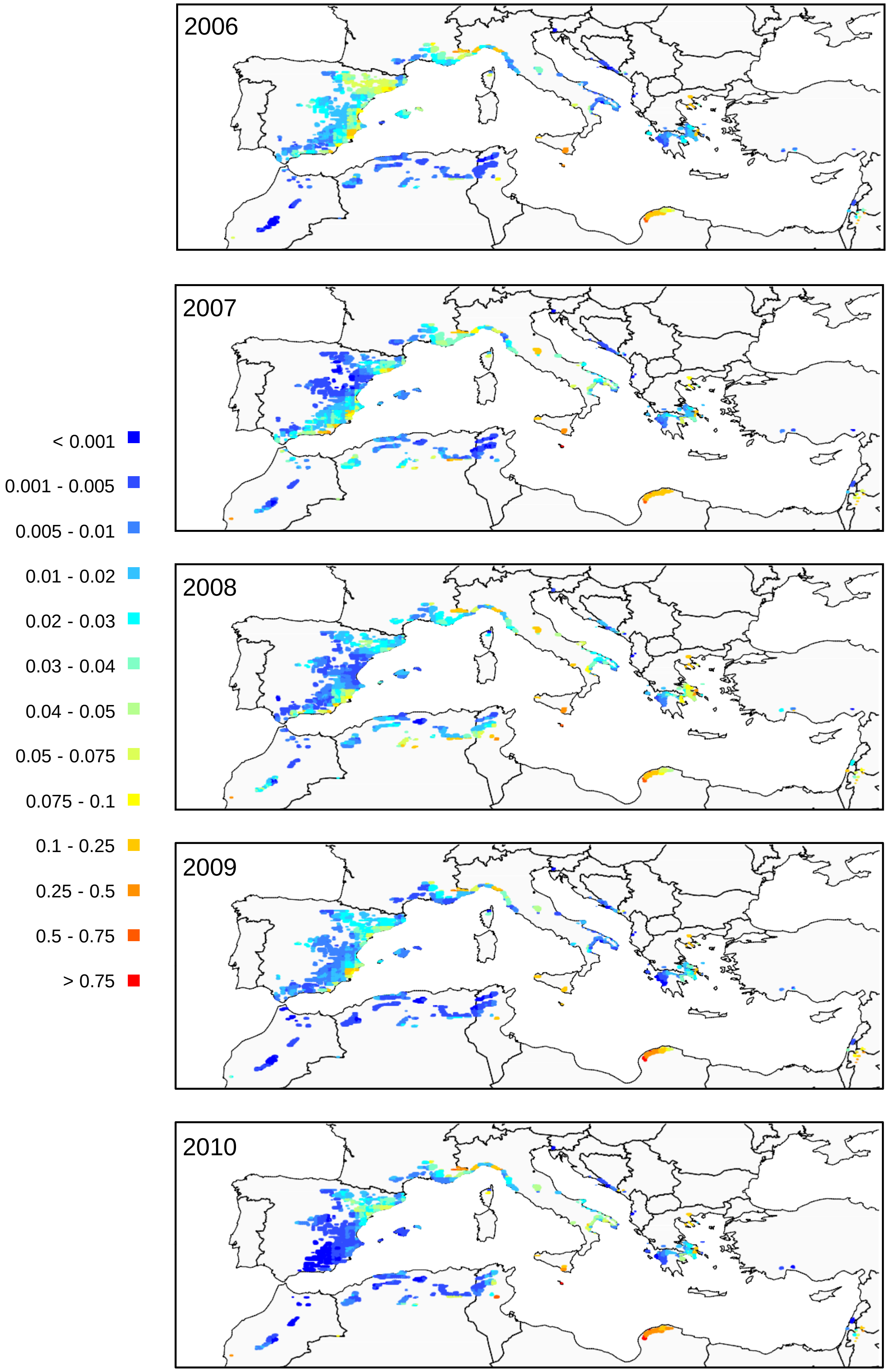

Predicted frequencies of MR across the distribution area of *Pinus halepensis* in the Mediterranean Basin (2006-2010).

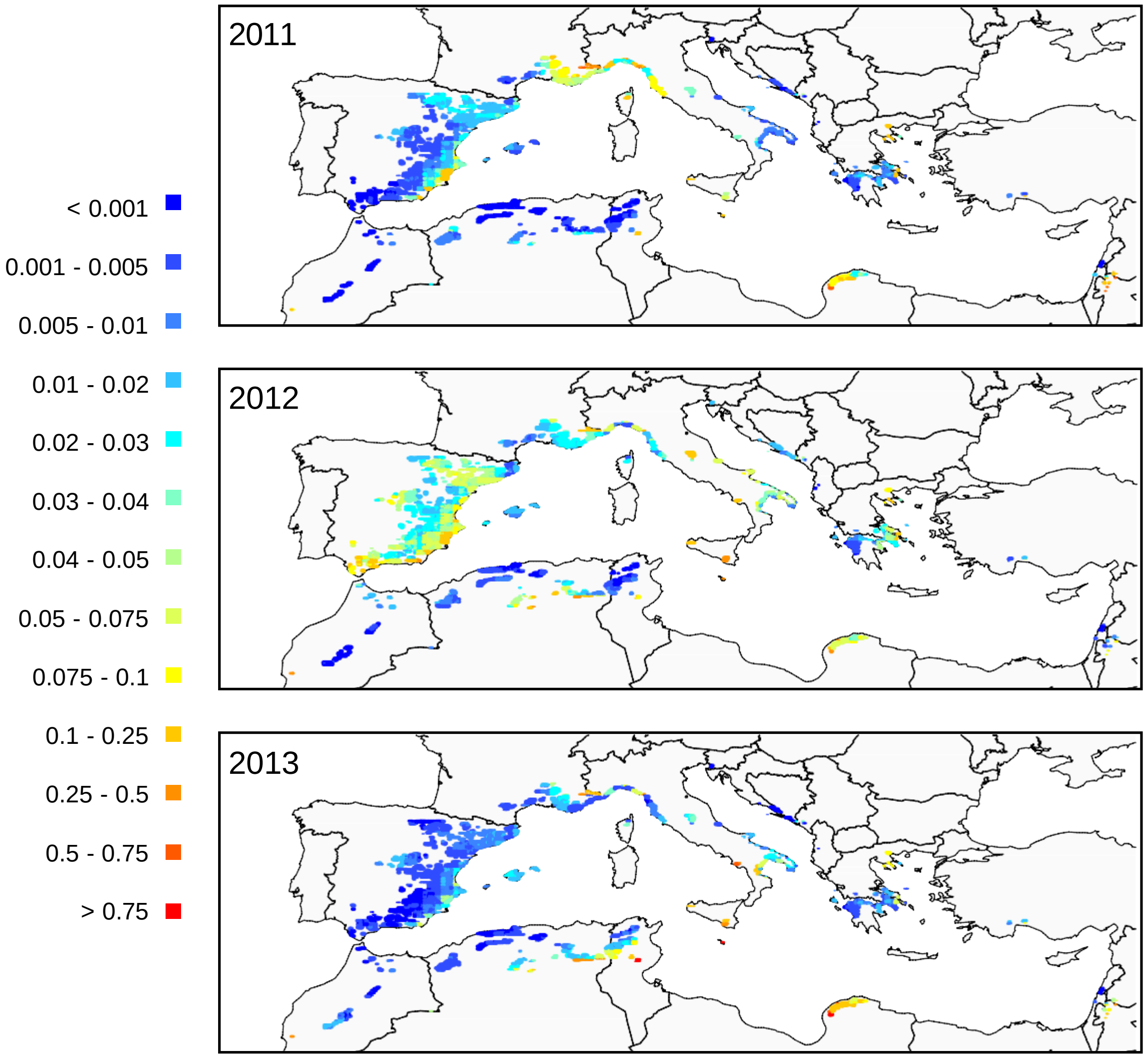

Predicted frequencies of MR across the distribution area of *Pinus halepensis* in the Mediterranean Basin (2011-2013).
